# The effect of 2′-fucosyllactose on simulated infant gut microbiome and metabolites; a pilot study in comparison to GOS and lactose

**DOI:** 10.1038/s41598-019-49497-z

**Published:** 2019-09-13

**Authors:** Krista Salli, Heli Anglenius, Johanna Hirvonen, Ashley A. Hibberd, Ilmari Ahonen, Markku T. Saarinen, Kirsti Tiihonen, Johanna Maukonen, Arthur C. Ouwehand

**Affiliations:** 1 DuPont Nutrition & Biosciences, Global Health & Nutrition Science, Kantvik, Finland; 2 DuPont Nutrition & Biosciences, Genomics & Microbiome Science, Madison, WI USA; 3 Vincit Oy, Turku, Finland

**Keywords:** Gastrointestinal models, Microbiome, Preclinical research

## Abstract

Human milk oligosaccharides (HMOs) shape gut microbiota during infancy by acting as fermentable energy source. Using a semi-continuous colon simulator, effect of an HMO, 2′-fucosyllactose (2′-FL), on composition of the infant microbiota and microbial metabolites was evaluated in comparison to galacto-oligosaccharide (GOS) and lactose and control without additional carbon source. Data was analysed according to faecal sample donor feeding type: breast-fed (BF) or formula-fed (FF), and to rate of 2′-FL fermentation: fast or slow. Variation was found between the simulations in the ability to utilise 2′-FL. The predominant phyla regulated by 2′-FL, GOS and lactose were significant increase in Firmicutes, numerical in Actinobacteria, and numerical decrease in Proteobacteria compared to control. Verrucomicrobia increased in FF accounted for *Akkermansia*, whereas in fast-fermenting simulations Actinobacteria increased with trend for higher *Bifidobacterium*, and Proteobacteria decrease accounted for *Enterobacteriaceae*. Short-chain fatty acids and lactic acid with 2′-FL were produced in intermediate levels being between ones generated by the control and GOS or lactose. In 2′-FL fast-fermenting group, acetic acid specifically increased with 2′-FL, whereas lactose and GOS also increased lactic acid. The results highlight specificity of 2′-FL as energy source for only certain microbes over GOS and lactose in the simulated gut model.

## Introduction

The gut microbiota of infants aged under 1 year is constantly changing and evolving^1^. Several factors, including diet, shape the microbiota of infants^2^. Human milk oligosaccharides (HMOs) are a structurally diverse group of carbohydrates with known health benefits and are highly abundant in and specific to human milk. HMOs are resistant to hydrolysis by intestinal enzymes^3^ and reach the colon, where they act as prebiotics. There, HMOs may promote the growth of beneficial bacteria, such as bifidobacterial species, which are often the predominate species in the microbiota of breast-fed (BF) infants, resulting in the production of microbial metabolites characteristic of carbohydrate fermentation^1,4^. Furthermore, HMOs resemble the structure of certain host epithelial cell surface glycans and thus may serve as soluble decoy receptors by preventing pathogen adhesion to epithelial surfaces and subsequent translocation^5^. HMOs improve host defence by modulating immunity and promoting intestinal barrier function^6^. Approximately 1% of ingested HMOs are absorbed into the infant’s systemic circulation and are excreted into the urine, where they also may contribute to anti-pathogen activity. However, most of HMOs are metabolised by the gut microbiota or excreted intact in the faeces of infants^7^.

HMOs are composed of five monosaccharides: glucose, galactose, N-acetylglucosamine, fucose and sialic acid. More than 100 HMOs have been identified that are differentially produced by lactating women, which indicates that the effects of HMOs on the infant gut microbiota varies, depending on the source^8^. The most abundant HMO in breast milk, that is currently also available via large-scale commercial production, is 2′-fucosyllactose (2′-FL)^9^, which selectively promotes the growth of bifidobacteria, mainly *Bifidobacterium longum* ssp. *infantis* and *Bifidobacterium bifidum*, but also stimulates growth of some *Bacteroides* species^10–12^. The concentration of 2′-FL is highest early in lactation during the first month postpartum^9,13^.

In this work, bacterial fermentation in the infant colon was modelled using the EnteroMIX® colon simulator^14–17^. Lactose is the predominant soluble digestible glycan in the milk; its primary role is to provide a readily available energy source to newborn mammals, whereas galacto-oligosaccharides (GOS) are included in infant formula to serve as prebiotics^18^. It is known that the abundance of HMOs affects the composition of the microbiota in infant faeces^19^, but very little is known about the effect of individual HMOs. The aim of our study was to compare 2′-FL to GOS and lactose with regards to their effects on the diversity, composition and metabolic activity of infant microbiota *in vitro*. The results were analysed combining all the simulations and by grouping the simulations according to the feeding type of faecal sample donor and 2′-FL fermentation velocities.

## Results

### Donor demographics

**Figure 1:**
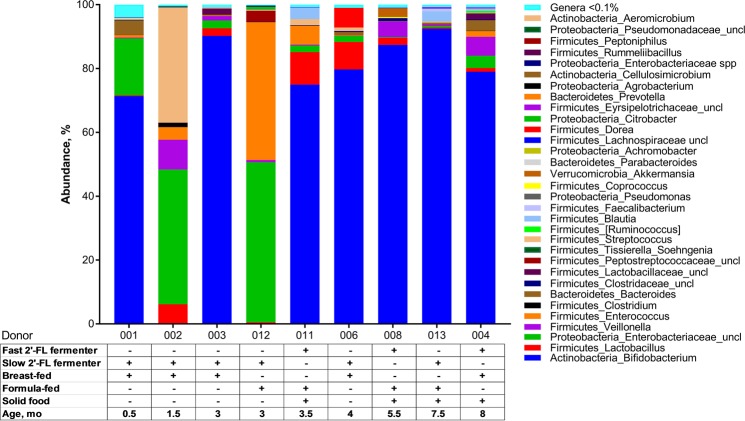
Genus level relative abundance of bacteria in each inoculum. Table below shows the donor demographics. 2′-FL = 2′-fucosyllactose.

Of the five BF donors, four were exclusively BF, and one was fed with formula rarely. The three FF donors used commercially available formula as their main source of food (Fig. 1). In Finland, donor breast milk is provided as the first source of nutrition in cases in which the mother is unable to breastfeed the new-born infant, making it difficult to find strict FF infants for studies. One BF donor and two FF donors had started on solid food (Fig. 1). One infant was delivered by caesarean section, and all but one used probiotic supplementation. Two of the donations (008 and 013) were received from the same infant at different ages (5.5 and 7.5 months, FF).

### Fermentation of 2′-FL during colon simulation

The utilisation of 2′-FL by complex bacterial communities varied between simulations (Table 1). When the amount of 2′-FL was analysed after simulations, three simulations (with inocula form donors 004, 008 and 011) metabolised 2′-FL quickly and completely early in the fermentation; additionally, fucose, a downstream metabolite, was initially detected at high levels, and then undetected. These three simulations are referred to as the 2′-FL fast-fermenting group. In the other six fermentations, 2′-FL was metabolised more slowly and was detectable at the end of fermentations. This change was accompanied with a slower rate of fucose generation (Table 1).

**Table 1:** 2′-fucosyllactose (2′-FL) amount in each vessel representing the different parts of the colon after the 48-h *in vitro* colon simulation.

a	2′-FL (%)/Vessel				b	Fucose (mg/l)/Vessel			
Donor	1	2	3	4	Donor	1	2	3	4
001	1.4	1.4	1.1	0.1	001	nd	nd	4	617
002	1.5	1.5	1.5	1.1	002	4	nd	1	5
003	1.5	1.5	1.3	0.8	003	46	35	34	33
004	0.1	nd	nd	nd	004	2972	2380	nd	nd
006	1.6	1.5	1.1	0.5	006	32	33	39	47
008	1.3	1.0	nd	nd	008	22	46	3	nd
011	1.5	0.1	nd	nd	011	86	1348	30	nd
012	1.4	1.4	1.0	0.6	012	31	31	115	119
013	1.5	1.5	1.4	1.0	013	30	59	30	31

The amount of (a) 2′-FL (%, w/v) and (b) fucose (mg/l) from each vessel are shown. nd = not detected.

### Microbiota composition by barcoded 16S rRNA amplicon sequencing

#### Microbial composition of the inocula originating from faecal sample used in the *in vitro* colon simulator

Total bacterial numbers were higher in inocula originating from infant faecal samples (faecal samples were mixed in artificial ileal medium and cultivated for 24 hours; see Materials and Methods) than samples from simulation vessels (Supplementary Table S1 and Fig. S3). During the simulation the microbial composition in each vessel evolves depending on the simulator conditions and carbon source. The samples taken after the simulation show endpoint of the resuscitation on the microbes that has taken place during simulation. 16S rRNA amplicon sequencing revealed a large heterogeneity in the microbial population of the inocula (Fig. 1). Bifidobacteria were not detected in the inocula of two donors (002 [BF] and 012 [FF]), which were slow-fermenters of 2′-FL.

#### Alpha and beta diversity

**Figure 2:**
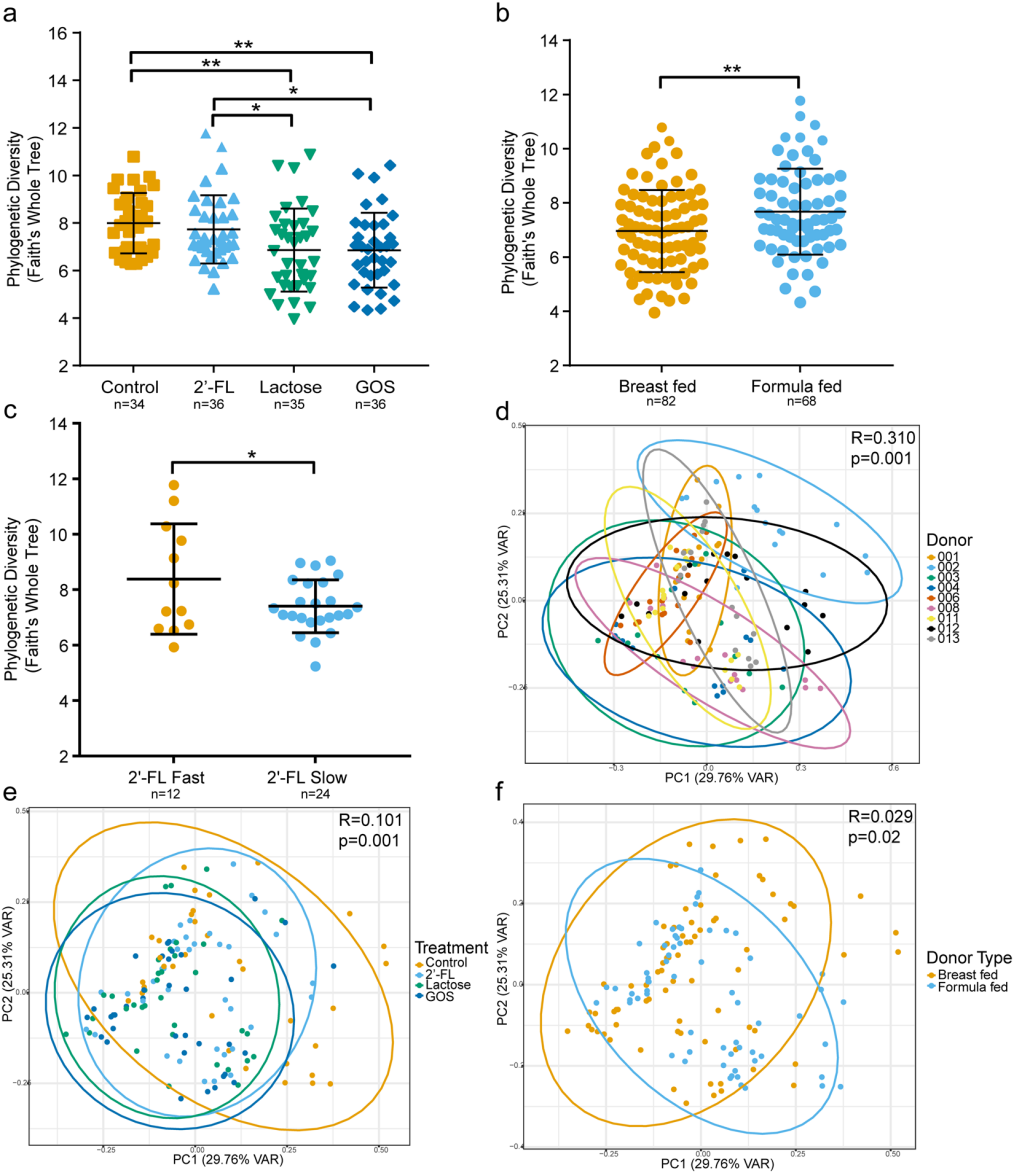
Alpha diversity (Faith’s Whole Tree metric) (**a**) between treatment groups from all samples, (**b**) between all breast-fed and formula-fed and (**c**) between 2′-fucosyllactose (2′-FL) fast- and slow-fermenting within the 2′-FL simulations. Beta diversity (weighted UniFrac metric) showing significant clustering of microbiota samples by (**d**) Donor, (**e**) Treatment, and (**f**) Donor feeding type from Analysis of Similarities (ANOSIM) test. ***p < 0.001, **p < 0.01, *p < 0.05. GOS = galacto-oligosaccharides.

Control and 2′-FL simulation samples contained significantly greater phylogenetic diversity than lactose and GOS simulation samples (p < 0.05, phylogenetic diversity whole tree metric) (Fig. 2a), which did not differ from each other. Furthermore, FF samples contained greater diversity than BF samples (p = 0.008; Fig. 2b), and similarly, fast-fermenting samples were more diverse than slow-fermenting samples (p = 0.04; Fig. 2c). Phylogenetic diversity increased towards the final vessels in all groups (Supplementary Fig. S1). Weighted UniFrac was used to assess pairwise sample dissimilarity, and the contribution of factors to sample clustering was tested using ANOSIM. Donor was the factor that contributed most to sample clustering (R = 0.310, p = 0.001), and the vessels from a particular donor clustered together in the PCoA (Fig. 2d). Treatment was the second most significant factor that contributed to sample clustering (R = 0.101, p = 0.001); vessels with 2′-FL clustered near the control fermentations, whereas GOS and lactose fermentations clustered more toward each other (Fig. 2e). Donor feeding type was the third most significant contributing factor (R = 0.029, p = 0.020) (Fig. 2f), whereas age was not statistically significant (data not shown).

#### Effect of treatment on microbial composition

**Figure 3:**
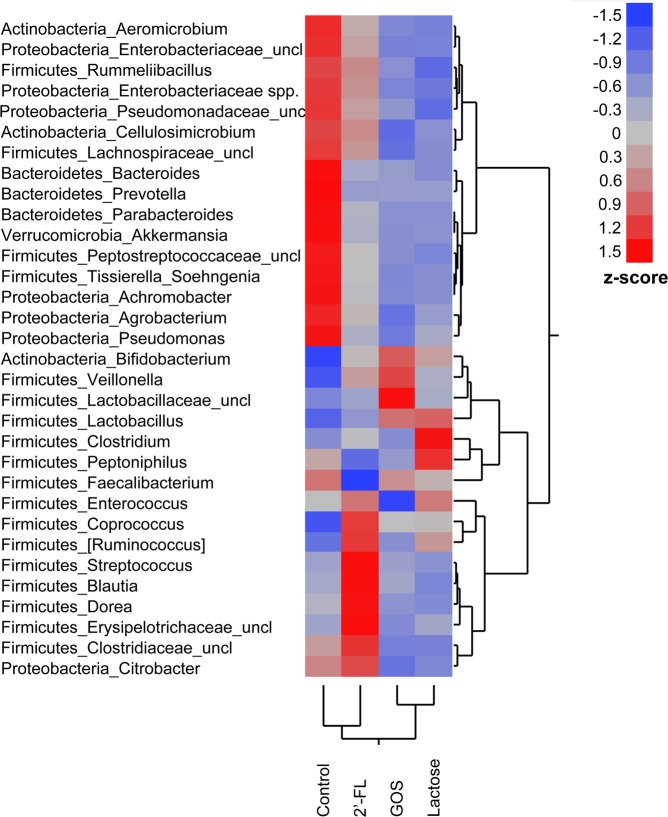
Heatmap with two-way hierarchical clustering of genus-summarised taxa abundance. Relative abundance was normalised across groups based on z-scores. 2′-FL = 2′-fucosyllactose, GOS = galacto-oligosaccharides.

Gut microbiota composition of all simulations was investigated, regardless of the donor feeding type or fermentation velocity, by dividing the simulations into BF and FF groups, and according to the 2′-FL fermentation velocity groups. The predominant phyla that were detected across all simulated infant microbiota samples were in average: Firmicutes (50% abundance), Actinobacteria (26%), Proteobacteria (18%), Bacteroides (5%) and Verrucomicrobia (0.3%). The detailed bacterial populations in each simulation of each vessel are shown in Supplementary Fig. S2. Cluster analysis based on genus-level abundance, normalised across the treatments, suggest that the microbiota compositions of the control and 2′-FL simulations resembled more to each other, and likewise, GOS and lactose were similarly grouped (Fig. 3). All treatments, regardless of the donor feeding type or 2′-FL fermentation velocities, when the vessels were combined together, increased the abundance of Firmicutes as compared with the control (Table 2). No other significant differences between the treatments were noted at the phylum level for 2′-FL; however, Proteobacteria abundance were decreased in lactose and GOS simulations. At the genus level, the most notable change for all three treatments compared to control was a significantly lower abundance of *Achromobacter* and *Pseudomonas* (Table 2). Otherwise, the effect that was exerted by 2′-FL shifted the microbiota in the same direction as with GOS and lactose but was not statistically significant.

**Table 2:** Phylum and genus-level microbiota changes with all the vessels combined among treatment groups, regardless of the donor feeding type or 2′-fucosyllactose (2′-FL) fermentation velocities. GOS = galacto-oligosaccharides.

Taxon	Abundance, % (mean ± SD)			Overall p-value	
	Control	2′-FL	Lactose	GOS	(FDR *adj*)^1^
Actinobacteria	20.7 ± 12.8	26.8 ± 19.9	27.8 ± 23.1	30.2 ± 22.1	0.53
*Aeromicrobium*	0.248 ± 0.43^a^	0.135 ± 0.29	0.023 ± 0.05^b^	0.031 ± 0.08^b^	0.027
*Cellulosimicrobium*	0.255 ± 0.21^a^	0.202 ± 0.22^a^	0.085 ± 0.12^b^	0.047 ± 0.05^b^	<0.001
Bacteroidetes	12.6 ± 17.0	2.80 ± 6.2	0.973 ± 2.2	1.90 ± 3.6	0.14
Firmicutes	35.0 ± 11.7^a^	48.8 ± 18.4^b^	60.0 ± 22.6^b^	57.2 ± 19.8^b^	<0.001
*Clostridiaceae_uncl*	1.95 ± 3.7^a^	3.03 ± 4.7^ab^	0.459 ± 1.2	0.445 ± 1.2^c^	0.008
*Enterococcus*	4.41 ± 4.9	5.12 ± 5.4^b^	5.07 ± 11.6^a^	3.17 ± 5.8^ac^	0.019
*Lachnospiraceae_uncl*	0.532 ± 0.79^a^	0.387 ± 0.57^a^	0.166 ± 0.40^b^	0.105 ± 0.22^b^	<0.001
*Lactobacillus*	9.0 ± 9.3^a^	15.2 ± 17.5^ab^	31.5 ± 28.7^b^	30.1 ± 26.5^bc^	<0.001
*Rummeliibacillus*	0.205 ± 0.38	0.160 ± 0.42^b^	0.031 ± 0.08^a^	0.063 ± 0.13	0.012
*Tissierella_Soehngenia*	2.30 ± 4.0^a^	0.797 ± 2.0	0.153 ± 0.80^b^	0.064 ± 0.26^b^	0.005
Proteobacteria	30.8 ± 17.5^a^	21.4 ± 16.1	11.1 ± 10.0^b^	10.7 ± 10.0^b^	<0.001
*Achromobacter*	0.794 ± 0.65^a^	0.283 ± 0.33^c^	0.091 ± 0.14^b^	0.067 ± 0.08^b^	<0.001
*Agrobacterium*	0.327 ± 0.45^a^	0.166 ± 0.21^a^	0.083 ± 0.16^b^	0.021 ± 0.02^b^	<0.001
*Citrobacter*	0.214 ± 0.29^a^	0.259 ± 0.47	0.083 ± 0.15^b^	0.063 ± 0.11^b^	0.002
*Enterobacteriaceae* spp.	0.186 ± 0.16^a^	0.148 ± 0.11	0.072 ± 0.07^b^	0.080 ± 0.08^b^	0.001
*Enterobacteriaceae_uncl*	27.7 ± 17.6^a^	19.9 ± 15.7	10.3 ± 9.6^b^	10.1 ± 9.6^b^	<0.001
*Pseudomonadaceae_uncl*	0.180 ± 0.41^a^	0.130 ± 0.23	0.052 ± 0.17^b^	0.078 ± 0.23^b^	0.004
*Pseudomonas*	0.907 ± 1.4^a^	0.358 ± 0.65^c^	0.333 ± 0.90^b^	0.154 ± 0.34^b^	<0.001
Verrucomicrobia	0.905 ± 3.2	0.212 ± 1.0	0.057 ± 0.02	0.039 ± 0.20	0.81

^1^Kruskal-Wallis tests with Benjamini-Hochberg false discovery rate (FDR) adjustments were conducted for main effect of treatment. Only genera with FDR < 0.1 are shown.

^a–d^Means within rows with different superscripts differ by p < 0.05 (Steel-Dwass Compare to Control post hoc test).

#### Effect of inocula donor feeding type on microbial composition

In comparisons of BF and FF, regardless of treatment or 2′-FL fermentation velocities, when the vessels were combined together, the only phylum that differed significantly was Verrucomicrobia (Table 3). Specifically, *Akkermansia* was more abundant in FF samples. In FF, *Lactobacillus* was the most prevalent and enriched genus compared with BF (Table 3). *Peptoniphilus* and *Prevotella* were virtually absent from FF samples, but none of the treatments was responsible for this effect.

**Table 3:** Phylum and genus-level microbiota changes with all the vessels combined between breast-fed and formula-fed infants regardless of treatment or 2′-fucosyllactose (2′-FL) fermentation velocities.

Taxon	Abundance, % (mean ± SD)		Overall p-value (FDR *adj*)
	Breast-fed	Formula-fed	
Actinobacteria	28.2 ± 23.1	24.4 ± 15.4	0.55
Bacteroidetes	3.69 ± 9.2	5.45 ± 11.2	0.31
*Prevotella*	0.285 ± 2.4	0.00 ± 0.0	0.067
Firmicutes	47.3 ± 21.3	54.1 ± 19.8	0.11
*Clostridiaceae_uncl*	0.799 ± 1.8	2.28 ± 4.3	**0**.**005**
*Dorea*	0.095 ± 0.30	0.243 ± 1.4	**0**.**027**
*Lachnospiraceae_uncl*	0.196 ± 0.40	0.414 ± 0.67	**0**.**044**
*Lactobacillaceae_uncl*	1.74 ± 5.5	0.179 ± 0.49	0.060
*Lactobacillus*	16.6 ± 22.0	27.5 ± 24.6	**0**.**005**
*Peptoniphilus*	0.208 ± 1.1	0.00 ± 0.0	**0**.**022**
*Peptostreptococcaceae_uncl*	0.585 ± 1.6	1.13 ± 1.6	**0**.**015**
*Streptococcus*	0.642 ± 2.1	0.018 ± 0.06	**0**.**022**
Proteobacteria	20.8 ± 18.2	15.4 ± 12.2	0.49
*Citrobacter*	0.072 ± 0.10	0.253 ± 0.41	**0**.**010**
Verrucomicrobia	0.002 ± 0.001	0.651 ± 2.43	**<0**.**001**
*Akkermansia*	0.002 ± 0.001	0.651 ± 2.43	**0**.**001**

^1^Kruskal-Wallis test with Benjamini-Hochberg false discovery rate (FDR). Only genera with FDR < 0.1 are shown. FDR < 0.05 are shown in bold.

At the phylum level, all treatments in FF group increased Firmicutes and decreased Proteobacteria compared with treatments in BF group; in BF, only GOS or lactose changed these phyla in the same direction as in FF (Supplementary Table S3). The increase of Firmicutes by lactose and GOS was attributed primarily to *Lactobacillus* (Supplementary Table S3). In BF, the genus that was changed only by 2′-FL was *Enterococcus* (member of the Firmicutes phylum) (Supplementary Table S3). Additionally, both 2′-FL and lactose, but not GOS, decreased the phylum Bacteroidetes in BF, which was explained by a decrease in the genus *Bacteroides*. Significant changes that were associated with 2′-FL in FF corresponded to *Achromobacter* and *Agrobacterium* (minor genera of the phylum Proteobacteria), which were decreased by all treatments, as well as *Coprococcus* from Firmicutes, which was enhanced by 2′-FL. The shift in *Achromobacter* was not dependent on grouping, as *Achromobacter* was also decreased by all treatments in BF. Grouping affected *Pseudomonas*, which was significantly reduced by all treatments in BF but not by 2′-FL in FF (Supplementary Table S3).

#### Effect of microbial composition to 2′-FL fermentation velocity

Comparisons of the 2′-FL fast- and slow-fermenting groups, regardless of treatment or the donor feeding type, with the data from all the vessels combined, showed that the 2′-FL slow-fermenting simulations had a higher abundance of the Proteobacteria phylum, which was explained largely by increased abundances of unclassified *Enterobacteriaceae* (Table 4). The phylum Actinobacteria was increased by the 2′-FL fast-fermenting group, with a trend toward higher abundance of *Bifidobacterium* (Table 4). At the genus level, the slow-fermenting group showed a significant increase in *Enterococcus* and a decline in *Coprococcus* compared with the fast-fermenting group, but otherwise, the changes were accounted for by minor genera (Table 4).

**Table 4:** Phylum and genus-level microbiota changes with all the vessels combined between 2′-fucosyllactose (2′-FL) fast- and slow-fermenter groups, regardless of treatment or the donor feeding type.

Taxon	Abundance, % (mean ± SD)		Overall p-value (FDR *adj*)^1^
	2′-FL Fast	2′-FL Slow	
Actinobacteria	36.9 ± 20.2	21.7 ± 18.0	**0**.**039**
*Bifidobacterium*	36.6 ± 20.3	21.1 ± 18.2	0.052
*Cellulosimicrobium*	0.096 ± 0.12	0.255 ± 0.20	0.085
Bacteroidetes	4.10 ± 5.4	2.15 ± 6.6	0.56
Firmicutes	54.5 ± 16.9	45.9 ± 18.8	0.23
*Clostridiaceae_uncl*	0.202 ± 0.26	4.44 ±± 5.3	0.095
*Coprococcus*	2.00 ± 2.0	0.107 ± 0.20	**0**.**001**
*Enterococcus*	1.93 ± 0.93	6.71 ± 6.0	**0**.**006**
*Erysipelotrichaceae_uncl*	0.088 ± 0.09	0.540 ± 1.6	0.082
*Lactobacillus*	23.9 ± 24.1	10.9 ± 11.5	0.085
[*Ruminococcus*]	1.61 ± 3.1	0.404 ± 2.0	**0**.**033**
Proteobacteria	4.34 ± 4.0	30.0 ± 12.7	**<0**.**001**
*Achromobacter*	0.129 ± 0.13	0.360 ± 0.40	**0**.**044**
*Citrobacter*	0.052 ± 0.11	0.363 ± 0.40	**0**.**003**
*Enterobacteriaceae* spp.	0.032 ± 0.04	0.206 ± 0.10	**<0**.**001**
*Enterobacteriaceae_uncl*	3.67 ± 3.3	28.0 ± 12.7	**<0**.**001**
*Pseudomonadaceae_uncl*	0.071 ± 0.20	0.160 ± 0.20	0.079
Verrucomicrobia	0.086 ± 0.20	0.275 ± 1.3	0.56

^1^Kruskal-Wallis test with Benjamini-Hochberg false discovery rate (FDR). Only genera with FDR < 0.1 are shown. FDR < 0.05 are shown in bold.

When the treatment effect was examined, in the presence of 2′-FL, Proteobacteria significantly decreased and Firmicutes significantly increased but only in the fast-fermenting group. In contrast, both GOS and lactose significantly decreased Proteobacteria abundance both in the fast- and slow-fermenting groups (Supplementary Table S4). Lactose significantly elevated Firmicutes in both groups, whereas Firmicutes was increased by GOS only in the slow-fermenting group. At the genus level, the changes in the Proteobacteria phylum by 2′-FL in fast-fermenting simulations were due to decreases in abundance of unclassified *Enterobacteriaceae* and certain minor genera, such as *Pseudomonas* and *Achromobacter* (Supplementary Table S4). The significant increase in Firmicutes in the fast-fermenting group was due to increases in *Coprococcus*, *Lactobacillus* and *Veillonella*; however, no statistical significance could be seen for these individual effects (data not shown). Lactose and GOS significantly increased *Lactobacillus* in the slow-fermenting group.

#### Total bacterial numbers and total bifidobacterial numbers

All treatments, regardless of the donor feeding type or 2′-FL fermentation velocity, resulted in significantly higher numbers of total bifidobacteria compared with control simulations, based on qPCR (2′-FL p = 0.023, lactose p = 0.009, and GOS p = 0.005). The effect of grouping on total bacteria (measured by flow cytometry) and total bifidobacteria (measured by qPCR) is shown in Supplementary Fig. S3. The number of total bacteria (p < 0.001) and total bifidobacteria (p < 0.001) increased from vessel to vessel, irrespective of groupings. When total bacteria were measured by flow cytometry, a systematic difference between the fast- and slow-fermenting types was observed, with greater increases in bacterial numbers across the vessels in the 2′-FL fast-fermenting simulations (p < 0.001) (Supplementary Fig. S3).

### Microbial metabolites

#### Metabolite data distribution

Short-chain fatty acids (SCFAs), lactic acid, branched-chain fatty acids (BCFAs), and biogenic amines (BAs) were measured to evaluate bacterial metabolism. Of the 19 metabolites that were measured, 9 were observed in over half of the samples (Supplementary Fig. S4). Results of 2-methyl-butylamine (0.01%), β-Phenyl-ethylamine (16.3%), and histamine (36.6%) are not reported, as they were detected in few samples, without any indication of the effects of treatment or feed type. Ethylamine was detected in 33.3% of samples, but only from inocula or simulation samples corresponding to BF donors.

#### Treatment effect on microbial metabolite production

**Figure 4:**
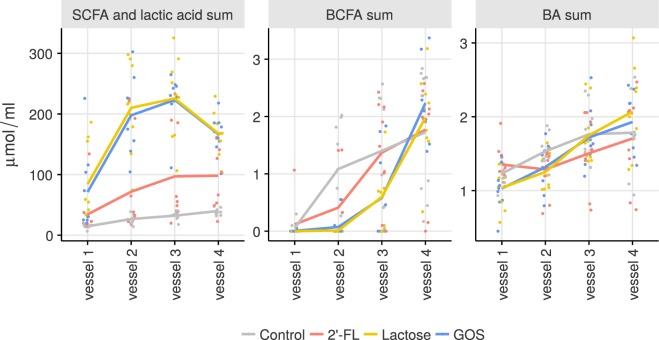
Smoothened averages of the metabolites measured from each vessel for all the simulations according the treatment. The dots are measurements from individual simulations. SCFA = short-chain fatty acids, BCFA = branched-chain fatty acids, BA = biogenic amines, 2′-FL = 2′-fucosyllactose, GOS = galacto-oligosaccharides.

The sum of SCFAs and lactic acid with 2′-FL increased significantly in the simulations: intermediate levels of these metabolites, between control and lactose or GOS, were observed (Fig. 4). Fermentation profiles also differed, decreasing toward the final vessel with GOS and lactose (Fig. 4, Supplementary Table S2). The differences between treatments were mainly due to acetic acid and lactic acid; with the former being significantly higher in all treatments (2′-FL p = 0.042, lactose p < 0.001, and GOS p < 0.001), and the latter only in lactose (p < 0.001) and GOS (p < 0.001) (Supplementary Fig. S5a).

Minor amounts of BCFAs were produced in the simulations, and consequently, only small differences were noted in the sum of BCFAs between treatments (Fig. 4). Significant changes were associated only with lactose and GOS, which had smaller levels of BCFAs in the early vessels (Supplementary Table S2 and Fig. S5b).

The sum of BAs (Fig. 4) showed the smallest difference between treatments, with no significant effects. Minor differences in individual BA levels were noted only for putrescine (lactose p = 0.035, GOS p = 0.046; Supplementary Fig. S5c). No other significant values were observed.

#### Effect of inoculum donor feeding type on microbiota metabolite production

**Figure 5:**
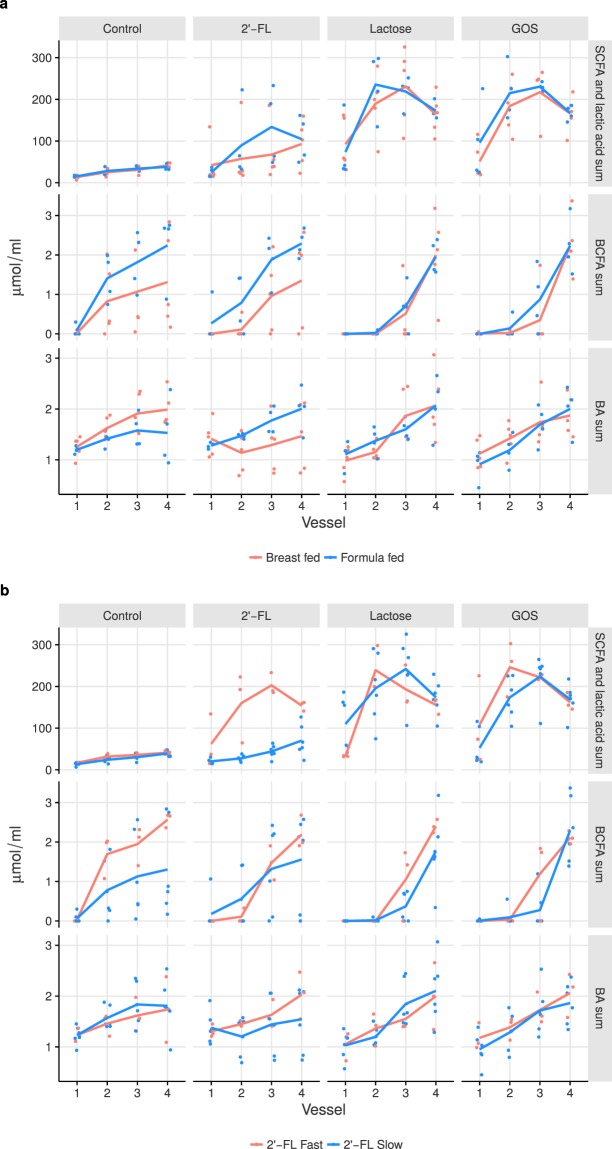
Smoothened averages of metabolites with simulations grouped by (**a**) breast-fed (BF) referring to simulations with breast-fed donors 001, 002, 003, 004 and 006 and formula-fed (FF) formula-fed donors 008, 011, 012, and 013, and (**b**) 2′-fucosyllactose (2′-FL) fast-fermenting, referring to simulations with donors 004, 008 and 011, or slow-fermenting referring to simulations with donors 001, 002, 003, 006, 012, and 013. The dots are measurements from individual simulations. SCFA = short-chain fatty acids, BCFA = branched-chain fatty acids, BA = biogenic amines, GOS = galacto-oligosaccharides.

In BF donor simulations, lactose and GOS but not 2′-FL significantly increased the sum of SCFAs and lactic acid, both in concentration and profile (Fig. 5a and Supplementary Table S2). This effect was due primarily to higher levels of acetic acid and lactic acid in lactose or GOS (acetic acid p < 0.001 and p = 0.002, lactic acid p = 0.004 and p = 0.020, respectively; Supplementary Fig. S6a). In simulations with FF inocula, 2′-FL elicited a significant increase in the sum of SCFAs and lactic acid (Supplementary Table S2), mainly due to an increase in acetic acid (p = 0.007), which was also enhanced by GOS (p = 0.022), but not lactose. Both GOS and lactose increased the levels of lactic acid (p = 0.003 and p = 0.020) in FF.

BCFAs were found later in simulation vessels, for both BF and FF with lactose and GOS (Fig. 5a and Supplementary Table S2). In addition, lactose decreased the concentration of total BCFAs in FF, which was attributed to a significant decline in isovaleric acid (p < 0.001).

In the BF group, lactose simulations decreased the sum of BAs (Supplementary Table S2), mainly due to lower putrescine levels (p = 0.006). 2′-FL also changed the BA profile, caused by lower cadaverine production earlier in the simulations (p = 0.020) (Fig. 5a and Supplementary Table S2). In the FF group, GOS effected a significant difference in the profile of BA production (Fig. 5a and Supplementary Table S2) due primarily to a decrease of spermine (p = 0.020). In addition, with FF, 2′-FL lowered spermidine levels (p = 0.020) and it was produced toward the end of the simulation (p = 0.024). Direct nonparametric comparisons between BF and FF groups revealed differences in individual BA production. Ethylamine was detected only in BF simulations (2′-FL p < 0.001, control p = 0.002, and lactose p = 0.015). With 2′-FL, cadaverine levels decreased in BF, whereas with FF donors, the levels rose (p = 0.04).

#### Effect of 2′-FL fermentation velocity on microbiota metabolite production

When the simulations were grouped according to 2′-FL fermentation velocities, 2′-FL in the fast-fermenting group was closer to lactose and GOS, based on the sum of SCFAs and lactic acid (Fig. 5b). Fast-fermenting 2′-FL and lactose and GOS significantly increased the sum of SCFAs and lactic acid, but differences in profile were noted only for lactose (Supplementary Table S2). A higher level of acetic acid was observed with the fast-fermenting 2′-FL (p = 0.003) and GOS (p = 0.020) treatments, accompanied by disparate fermentation profiles of butyric acid (p = 0.046) and valeric acid (p = 0.011) by 2′-FL and propionic acid by GOS (p < 0.001; Supplementary Fig. S6b). For the slow-fermenting simulations, increases in SCFAs and lactic acid were observed only with lactose and GOS (Fig. 5b and Supplementary Table S2), which were due to a rise in acetic and lactic acid (acetic acid p < 0.001, and p = 0.004, lactic acid p = 0.003, and p = 0.006, for lactose and GOS, respectively; Supplementary Fig. S6b).

In fast-fermenting simulations, the levels of total BCFAs were significantly decreased by all treatments (Fig. 5b and Supplementary Table S2), due to reduced levels of 2-methylbutyric acid in 2′-FL (p = 0.020) and GOS (p = 0.022), whereas lactose only increased isobutyric acid (p = 0.017) late in the simulations. In the slow-fermenting simulations, lactose and GOS significantly increased total BCFA concentrations but only in later vessels (Supplementary Table S2). Overall, less BCFAs were produced, which was attributed primarily to lower production of isovaleric acid (GOS p = 0.009; lactose p = 0.022).

There were no significant changes in BAs between the fast- and slow-fermenting groups (Fig. 5b and Supplementary Table S2). With slow 2′-FL fermenting group GOS simulations, there was less putrescine (p = 0.019), and in fast-fermenting group GOS simulations, spermine was produced late in the simulations (p < 0.001).

## Discussion

In this study, nine *in vitro* colon simulations were performed with inocula originating from faecal samples from infant donors aged under 1 year. The effect of 2′-FL on the composition of simulated infant microbiota and on the production of microbial metabolites was compared with GOS and lactose. Several parameters from *in vitro* fermentations were measured to evaluate the prebiotic characteristics of the treatments: total bifidobacterial numbers, microbiota composition, and the predominant microbial metabolites. The EnteroMIX® colon simulation model simulates various stages of fermentation, proceeding from the proximal to the distal colon. The simulations were run 48 hours with substrate feeding every 3 hours to adapt microbiota to the particular substrate prior the analysis of the microbiota composition and metabolites. Clear benefit for utilisation of a simulation model is that it enables studying metabolite production by complex microbial ecosystem while lacking the absorption by the intestinal epithelium; measuring from the faecal samples does not reflect *in situ* production of metabolites^20^. It also enables studying the colonic fermentation, which is difficult to study *in vivo* from human beings.

Previously, the EnteroMIX® colon simulation model has been used to study the effects of probiotics and prebiotics using adult faecal sample donors^14–17^, and the study here is the first to describe its’ usage for modelling infant colonic fermentation. Although it has been used to model the adult colon^16^, additional validation is likely to be needed to determine its usability for infant studies. Nonetheless, we determined the effects of 2′-FL in all simulations and by data subgrouping, according to the feeding regimen of the faecal inoculum donor, to BF and FF, and according to the velocity by which 2′-FL was fermented during the simulation, to 2′-FL fast- and slow-fermenting. There were vast differences between simulations in the ability of the complex microbiota to consume 2′-FL, and these changes were not explained by donor demographics.

One source of heterogenicity between simulations arose from donor samples. Certain bacteria, such as bifidobacteria, can be sensitive to freezing and thawing, and therefore, the storage of the samples—initially at −20 °C and then at −80 °C—may have caused variation in this study. As infant donors can only donate a small amount of faecal material at a given time or even each day, freezing and combining of faecal samples was done to ensure same microbiota conditions for the control, 2′-FL, GOS and lactose simulations. However, some studies have reported variability in the number of bifidobacteria detected from infant faeces^21^. The variability might decrease by selecting more homogenous donors—e.g., by eliminating the effect of solid food—which could improve the model.

Various factors affect the composition of an infant’s microbiota, such as delivery method (vaginal or caesarean); age; diet (breast milk or infant formula); the use of antibiotics, probiotics or other medication; maternal HMO secretion type and the environment and lifestyle of the family^2,22–26^. It is the cessation of breast-feeding rather than the introduction of solid food that defines composition of the microbiota in infants^27,28^. Also, in our study, large heterogeneity in microbiota composition of the infant faecal based inocula samples was observed as reported^29,30^. Likewise, in a recent study by Wiese *et al*. (2018), who used a similar but single-vessel colonic model, variation between two male infants was found^31^. In another study, the abundance of bifidobacteria was shown to be lower in Finland and Estonia than in Russian Karelia with *B*. *bifidum* and *B*. *longum* being more prevalent in Russians and *B*. *breve* more prevalent in the Finnish cohort^32^. In the future, it would be important to determine whether the changes in the composition of the bifidobacteria occur with infant-type *B*. *longum* ssp. *infantis* (typical of BF infants) or with other species, such as *B*. *breve*, *B*. *adolescentis*, *B*. *longum* ssp. *longum* and *B*. *bifidum*, that more common in FF infants^21,28^. Therefore, in this study, although the prevalence of bifidobacteria was high in the most of inocula, irrespective of the major feeding type of the donor, at species level differences might have been detected. Bifidobacterial species have differential abilities in degrading HMOs, including 2′-FL. Specifically, *B*. *longum* ssp. *infantis* utilises HMOs to completion, whereas other bifidobacteria partially utilise HMOs or not at all^11,33,34^.

Gut microbial diversity increases gradually after birth^2,27,35^. In our study, 2′-FL, both overall and in the FF and fast-fermenting groups, generated a more diverse microbiota after the simulations than did GOS or lactose, or grouping to BF or slow-fermenting groups. BF infants harbour less diverse microbiota compared with FF infants, primarily due to a greater proportion of bifidobacteria and lactobacilli in BF infants versus FF infants^21,27,28^. Although this lower microbial diversity is associated with breast-feeding and the prevalence of beneficial bifidobacteria, microbial diversity has dual role: reduced diversity of infant microbiota is linked to various diseases (in childhood and later in life), such as colic, necrotising enterocolitis, eczema, asthma, diabetes, and autism^1,21,36–39^. The increase in microbial diversity with 2′-FL compared with GOS and lactose, and fast-fermentation versus slow-fermentation, indicates the differential ability of 2′-FL and fast-fermentation to promote the growth of microbes that are metabolically capable of utilising 2′-FL efficiently in the gut modelling conditions.

Not all HMOs lead to the same changes in the composition or activity of the gastrointestinal microbiota or have the same benefits upon host well-being and health^11^. The structure of an HMO determines its prebiotic effect, which is dependent on the metabolic capability of a bacterium. In addition, microbial communities likely utilise cross-feeding to sequentially degrade carbohydrates^40,41^. The only available clinical study on the changes in microbiota after consuming two different HMOs over a two-week intervention period was performed in an adult population^42^. Both 2′-FL and lacto*-N-*neotetraose, alone and the combination, increased Actinobacteria, mainly *Bifidobacterium* spp.^42^. In the current study, we did not expect that 2′-FL alone as an carbon source would be able to benefit the entire bifidobacterial community. Substantial selection occurred, indicating that 2′-FL alone promoted the growth of bacteria that can metabolize it and that the maintenance of the entire community structure requires a more complex mixture of HMOs. However, when analysed by qPCR, all treatments increased total bifidobacterial number over the control simulation, which is an indication of the bifidogenic effect that has been observed for GOS^18^ and for 2′-FL^11,12,43^. Yet, by 16S sequencing, we could merely observe a trend toward increasing amounts of bifidobacteria in 2′-FL fast-fermenting simulation samples, but this might be due to methodological difference in terms of relative measurement by 16S sequencing and absolute quantification by qPCR^44^.

Notably, the overall changes at the phylum level, across all feed and 2′-FL fermentation groups, were similar: both GOS and lactose decreased the overall abundance of Proteobacteria and increased the relative abundance of Firmicutes compared with the control fermentation. When the overall changes of the microbiota were followed, the effects of 2′-FL often followed those of GOS or lactose, but less extensively. These results correlated with the metabolite data. The lower ability of 2′-FL to act as a substrate and promote microbiota growth is an indication of the selectivity of 2′-FL over GOS and lactose. In pure bacterial cultures, GOS ad lactose, are supporting the growth of wider range of microbes compared to 2′-FL (manuscript in preparation).

Certain bacteria were affected by all three treatments. For instance, we observed that 2′-FL, GOS and lactose decreased the abundance of the pathogens *Achromobacter* and *Pseudomonas*^45,46^, which are higher in colic^38^. The effect of *Achromobacter* did not depend on the type of feeding, as *Achromobacter* was affected similarly by the different treatments in the BF or FF simulations. The higher abundance of *Lactobacillus* in FF versus BF was unexpected as *Lactobacillus* normally associates with BF^32^. This change probably reflects the robust effect of GOS and lactose in promoting *Lactobacillus* growth, as it was increased significantly by both treatments in FF. No single treatment explained the increase of *Akkermansia* in FF, which is higher in infants with eczema^47^. However, virtually no *Akkermansia* were detected by sequencing in any of the treatments in the BF group, whereas in FF, *Akkermansia* were observed in FF but without any significant treatment effects. It is believed that due to its ability to degrade mucin, *Akkermansia* functions in reducing the integrity of the intestinal barrier and in the penetration of allergens through the intestinal wall^47^. There were other, more specific changes in the microbiota that are associated with eczema as 2′-FL increased the genus *Coprococcus* spp. in FF. *Coprococcus* species ferment carbohydrates and produce butyrate^48^, and increases in *Coprococcus eutactus* have been shown to associate with decreased eczema severity^39^. In BF donor simulations, 2′-FL was the only treatment that enhanced the relative abundance of *Enterococcus* spp. and lowered *Bacteroides* spp. in comparison to control simulations. Distinct *Enterococcus faecalis* strains have a limited capacity for growth on isolated HMOs^49^ and in 2′-FL^12^, but growth in an isolated system does not always reflect the complex ecosystem of gut microbiota. *Enterococcus* spp. are usually detected at higher abundance in FF versus BF infants^50,51^. Enterococci contain putative pathogenic bacterial strains due to the acquisition of antibiotic resistance genes^52^, but enterococci also harbor beneficial bacterial strains that can alleviate inflammatory responses^53,54^. In general, BF infants possess lower levels of *Bacteroides* spp. compared with FF infants^55–57^ and the introduction of solid foods can increase this genus^57,58^, here due to relatively low number of simulations, however, this effect was not seen.

In the study by Matsuki *et al*. 2016, in infant faecal samples the abundance of bifidobacterial population was associated with increased organic acid concentrations and decreased pH values of feaces^30^. Our results are consistent with previous results on major SCFA and lactic acid production. SCFA levels differ in BF and FF infants with higher levels of faecal SCFAs in FF compared to BF^59^. Acetate is commonly more prevalent in BF infants, with nearly complete absence of butyrate^59^, and fucosyllactose-utilising bifidobacteria were in defining role in acetate production when bifidobacterial isolates from infant faecal samples were investigated^30^. Our study is different to the study by Matsuki *et al*. 2016 as a simulated microbial ecosystem was studied here^30^. Furthermore, when the results were grouped according to feeding type to FF or according fast-fermenting, 2′-FL was found to increase the acetate production, indicating 2′-FL is having a supporting role in the metabolism of bacteria capable of producing acetate, even though no clear increases in the bifidobacterial populations were noted in our study. In addition to acetate, propionate and butyrate are higher in FF infants^59,60^. Lactate is also commonly detected in the faeces of infants, and reduces the faecal pH^60^. Fructo-oligosaccharide utilisation with a four-species consortium of bacteria effected a higher prevalence of lactobacilli and greater lactate levels than 2′-FL^61^. Similarly, in the current study, significantly more lactic acid was produced with more readily fermentable carbon sources, GOS and lactose than with more specifically fermented 2′-FL, correlating with an increase in *Lactobacillus* spp. by GOS and lactose. In our earlier colon simulation study with GOS and inocula from adult faecal sample, little lactic acid was produced^15^, whereas in the current study, GOS elicited significantly more lactic acid than the control. Simulations with adult inocula produce some lactic acid with 2% lactose^17^, but in this work with infant inocula, increased lactic acid production during simulations was observed. In published BF infant faecal inoculum batch-fermentation study, acetic acid was produced from various carbohydrates^62^, as has also been found for GOS in both BF and FF fermentations^10^. In our model, acetic acid and lactic acid levels were significantly increased by GOS regardless of the feeding type and by lactose in BF donor simulations, whereas in FF donor simulations, only lactic acid was enhanced by lactose. With 2′-FL, a significant increase in the level of acetic acid was observed with the FF group. Furthermore, in the 2′-FL fast-fermenting simulations, more acetic acid but little lactic acid was produced. There was no difference in lactic acid production between the fast- and slow-fermenting groups. FF fermentations have earlier been shown to produce also more propionic acid and butyric acid with various carbohydrates than with BF fermentations^62^. In the current study, only 2′-FL effected a significant change in propionic acid production in FF samples; also, GOS and lactose increased the amount of propionic acid. However, due to the high variance and the low number of simulations, these effects by GOS and lactose were not statistically significant.

It has been proposed that increased production and utilisation of lactate by lactate-utilising H_2_-producing bacteria (to prevent toxic lactate accumulation) contribute to colic symptoms^63^. In addition, SCFAs may induce osmotic diarrhoea, which is linked to excess production and limited colonic absorption of SCFAs^64^. The ability of GOS to generate larger amounts of SCFAs compared with 2′-FL has been reported^10,65^. However, as we evaluated the effect of only one HMO on the composition of the complex fermentation system—not defined bacterial species and metabolites—the combined effect of multiple HMOs can be different.

There were minor changes in BA production by 2′-FL: decreased cadaverine in BF, and less spermidine in FF, due to changes in amino acid degradation of the microbiota. Although there is accumulating evidence of the function of BA in intestinal signalling, there remain little data on the specific health benefits in infants^66^.

In conclusion, although the inocula from infant donors introduce variability in fermentation simulation studies, it provides opportunities not possible *in vivo*. This *in vitro* model is an alternative enabling better understanding of the effects of 2′-FL, GOS and lactose on microbial composition and metabolism. In our study, 2′-FL, as well as GOS and lactose promoted the growth of bifidobacteria. The slight changes in microbiota caused by 2′-FL were reflected by the intermediate production of SCFAs with lower production of acetate and lactate compared with lactose or GOS. There were also donor differences in the ability of the to ferment 2′-FL, indicating that 2′-FL fermentation requires more specific microbial activity than lactose or GOS fermentation. By modelling the infant gut fermentation, the need to conduct animal trials decreases, and they may provide direction for clinical studies.

## Material and Methods

### Colon simulator model

For modelling the infant gut microbiota, frozen infant faecal samples were used to prepare an inoculum of the colon simulator system. Donor infants, all aged under 1 year, were in good health and had not been medicated with antibiotics. A parent of each infant gave informed consent and provided background information on the infant who was donating the faecal sample (age, food, supplements, allergies, and delivery mode). Parents were provided with the instructions and equipment for sample handling. Infant faecal samples were frozen immediately at home before the samples were collected from each family. The study was reviewed and approved by the Coordinating Ethical Committee of the University of Helsinki (Decision number 139/13/03/00/16). All methods were in accordance with the national guidelines in Finland.

To study the effect of 2′-FL on the infant intestinal microbiota, the four-stage semi-continuous EnteroMIX® colon simulator model was used^14,16,17^. The technical specifications of this *in vitro* model have been described earlier^16,67^. In brief, the simulator consists of four units, in which four simulations from the same inoculum can be run simultaneously and in parallel. A single unit of the EnteroMIX® colon simulator contained four sequentially connected glass vessels, V1 to V4, representing the different compartments of the human colon, ranging from the ascending colon to the sigmoid/rectum area. The volume of microbial slurry increased from V1 (6 ml) to V4 (12 ml) to mimic reduced flow. pH levels (pH 5.5, 6.0, 6.5 and 7.0 for vessels V1, V2, V3, and V4, respectively) were controlled and adjusted with 1% ammonia. The entire system was maintained at 37 °C in an anaerobic atmosphere. All run parameters were controlled with a computer using customised software. Artificial ileal fluid was used as a medium in the EnteroMIX® colon simulator^67^. 2′-FL (DuPont Nutrition and Health, Kantvik, Finland and Inbiose, Ghent, Belgium), lactose (Sigma-Aldrich, St. Louis, MO, USA) and GOS (Clasado Biosciences, St Helier, Jersey, United Kingdom) were suspended in artificial ileal fluid (2% concentration, w/v) to serve as the sole carbon source. Artificial ileal fluid alone was fed to the system for control simulations.

Parents froze the collected faecal samples at −20 °C, and the samples were stored at −80 °C in the laboratory until used as inoculum. When required to generate enough inoculum for the four units of the simulator, faecal samples from the same donor collected within one week were pooled. The faecal samples from a single donor were mixed with 3 parts (wt/wt) of artificial ileal fluid, filtered through 0.3 mm metal mesh and incubated anaerobically at +37 °C for 24 hours, and then, this faecal slurry was added to the simulator units. Samples from the slurry were taken to determine the composition of the inoculum. The test products (2′-FL, GOS, lactose) or control were fed into the simulator system at 3-hour intervals during the simulation for a total time of 48 hours. Samples were then collected from the simulator vessels; the composition of the simulated microbiota, and microbial metabolites were analysed.

### Quantitative polymerase chain reaction (qPCR)

DNA from the colon simulation samples was extracted using the MagMAX™ with Total Nucleic Acid Isolation Kit (Ambion Inc., Austin, TX, USA) and the Mag MAX ™ Express 96 sample preparation system (Life Technologies, Halle, Belgium) according the manufacturers’ instructions. Bead beating was performed with a Precellys24 homogenizer (Bertin Technology, Montigny le Bretonneux, France). DNA was further purified with the One-Step-96^TM^ PCR Inhibitor Removal Kit (Zymo Research, Irvine, CA, USA), and the amount of DNA was determined using a Qubit® 3.0 Fluorometer (Thermo Fisher Scientific, Waltham, MA, USA). Total bifidobacteria were quantified by real-time quantitative polymerase chain reactions (qPCR) using TaqMan and Applied Biosystems Real-Time PCR equipment and software (ABI 7500 FAST, Applied Biosystems, Foster City, CA, USA), as described^16,68^. Standard curves, consisting of 10-fold dilutions of target species DNA, were used for quantification.

### Microbial composition by barcoded 16S rRNA amplicon sequencing

The V4 variable region of the 16S rRNA gene was PCR-amplified from donor inoculum samples and control, 2′-FL, lactose, and GOS simulations samples, as described^69^. The amplicon pool was sequenced using the Illumina MiSeq system with 2 × 250 bp reads (DuPont Pioneer, Johnston, IA, USA) and analysed using the Quantitative Insights Into Microbial Ecology pipeline (QIIME v. 1.9.1)^69,70^. Sequences were clustered into operational taxonomic units (OTUs) at 97% sequence similarity against the Greengenes database (v. 13.8)^71^. Taxa compositions are reported as relative abundance (% of total sequences) and were visualised using Prism (GraphPad Software, v. 7.0, La Jolla, CA, USA).

### Analysis of microbial metabolites

The concentrations of SCFAs, BCFAs, and lactic acid from colon simulation samples were analysed using chromatographic methods, as described by Ouwehand *et al*.^72^. BAs from samples were analysed as dansyl derivates by reversed-phase high-performance liquid chromatography, as described by Saarinen^73^.

### Quantification of fucose and 2′-FL

Standard solutions of fucose (Sigma-Aldrich, St. Louis, MO, USA) and 2′-FL (DuPont Nutrition and Health, Kantvik, Finland and Inbiose, Ghent, Belgium), were prepared in water to concentrations of 40, 30, 20 and 10 mg/l and stored at +4 °C. Sample solutions were centrifuged at 16,000 × g for 5 min; then, 50 µl of the supernatant and 200 µl of ethanol were mixed in a microcentrifuge tube and incubated at +4 °C for 30 min. After centrifugation at 16,000 × g for 5 min, 200 µl of the supernatant was evaporated to dryness at 70 °C on a vacuum centrifuge, and the solid residue was dissolved and diluted in water and filtered. High-performance anion-exchange chromatography of fucose and 2′-FL was performed on a Dionex DX600 system that consisted of a GP50 gradient pump, an ED50 detector that was equipped with a working gold electrode, and a combined pH-AG/AgCl reference electrode, and an AS50 autosampler (Dionex, Sunnyvale, CA, USA). For the separation, a CarboPac PA1 analytical column (250 × 4 mm, Dionex, Sunnyvale, CA, USA) and a precolumn (50 × 4 mm) of the same material were used. The mobile phase consisted of: A (water) and B (200 mM NaOH). The following gradient was used for the separation: 0–6 min., A = 97% and B = 3%; 6–20 min., a linear decrease of A to 40% and a linear increase of B to 60%; 20–24.5 min., A = 40% and B = 60%; 25–33 min., A = 0% and B = 100%; 34–46 min., A = 3% and B = 97%. The mobile phase flow rate was 1 ml/min, and the injection volume was 25 µl. For the detection of the analytes, 300 mM NaOH was added to the eluent post-column through a mixing tee at a rate 0.6 ml/min, and the following potential-time sequence was used for amperometric detection: 0.05 V (0–0.40 sec.), increase to 0.75 V (0.40–0.41 sec.), 0.75 V (0.41–0.60 sec.), decrease to −0.15 V (0.60–0.61 sec.), and −0.15 V (0.61–1.00 sec.). The retention time of fucose and 2′-FL was 6.3 min. and 22.8 min., respectively.

### Statistical analysis

Alpha diversity comparisons were calculated within the QIIME for the Phylogenetic Diversity (PD) Whole Tree metric^74^ using an OTU table, rarefied at a sequence depth of 11,206. Non-parametric t-test using 1000 Monte Carlo permutations and Benjamini-Hochberg false discovery rate (FDR) correction were used for pairwise comparisons^75^. Beta diversity was calculated using weighted UniFrac^76^, and the contribution of individual factors was tested with Analysis of Similarities (ANOSIM) in QIIME. For the ANOSIM test, the R value represents effect size and is constrained between -1 and 1, where a value of zero indicates random grouping. The closer the R value is to 1, the greater the effect that individual factor contributes to sample clustering. Distance matrices were visualised using principal coordinates analysis (PCoA) with the R (v. 3.4) *ggplot2* package^77,78^. Differentially abundant taxa (>0.1% abundance) were determined by Kruskal-Wallis test, and p-values were adjusted by FDR. Post hoc comparisons were conducted with Steel-Dwass Compare to Control test. A hierarchical clustered heatmap, based on taxa abundance, was generated by two-way analysis using Ward’s minimum variance method, wherein each taxon was normalised across treatments, based on z-scores (JMP Pro, v.13, SAS Institute, Cary, NC, USA).

Longitudinal data across multiple vessels were analysed using the nonparametric and robust methods that were developed by Brunner and colleagues^79^. These methods make minimal assumptions of the shape and distribution of the observed curves and are unaffected by extreme outliers, rendering these methods ideal for the analysing the collected simulation data. These approaches can assess the statistical significance of differences in levels between groups of interest and in the shape of the curves. These methods are part of the R package *nparLD*^80^. p-values from multiple simultaneous tests were corrected for FDR using the Benjamini-Hochberg method^75^. p-values of 0.05 or less were considered statistically significant. The effects of 2′-FL fermentation velocity and feed grouping on the numbers of bifidobacteria and total bacteria during the simulations were analysed using a parametric model. Specifically, a linear mixed effects model was used, with random intercepts and slopes for the subjects and vessel number coded as a continuous covariate. A fixed second-order slope was included to account for nonlinear growth of bacteria in the vessels. For model selection, statistically non-significant interaction terms were excluded to obtain increased power for the estimation of the parameters of interest.

## Supplementary information

Supplementary tables and figures

## References

[1] Milani, C. *et al*. The First Microbial Colonizers of the Human Gut: Composition, Activities, and Health Implications of the Infant Gut Microbiota. *Microbiol*. *Mol*. *Biol*. *Rev*. **81**, 10.1128/mmbr.00036-17 (2017). 10.1128/MMBR.00036-17 PMC5706746 29118049

[2] Chong Clara, Bloomfield Frank, O’Sullivan Justin (2018). Factors Affecting Gastrointestinal Microbiome Development in Neonates. Nutrients.

[3] Engfer MB, Stahl B, Finke B, Sawatzki G, Daniel H (2000). Human milk oligosaccharides are resistant to enzymatic hydrolysis in the upper gastrointestinal tract. Am. J. Clin. Nutr..

[4] Smilowitz JT, Lebrilla CB, Mills DA, German JB, Freeman SL (2014). Breast milk oligosaccharides: structure-function relationships in the neonate. Annu. Rev. Nutr..

[5] Newburg DS (2005). Innate immunity and human milk. J. Nutr..

[6] Chichlowski M, De Lartigue G, Bruce German J, Raybould HE, Mills DA (2012). Bifidobacteria isolated from infants and cultured on human milk oligosaccharides affect intestinal epithelial function. J. Pediatr. Gastroenterol. Nutr..

[7] Bode L (2012). Human milk oligosaccharides: every baby needs a sugar mama. Glycobiology.

[8] Kobata A (2010). Structures and application of oligosaccharides in human milk. Proc. Jpn. Acad. Ser. B Phys. Biol. Sci..

[9] Thurl Stephan, Munzert Manfred, Boehm Günther, Matthews Catherine, Stahl Bernd (2017). Systematic review of the concentrations of oligosaccharides in human milk. Nutrition Reviews.

[10] Vester Boler BM (2013). *In vitro* fermentation characteristics of select nondigestible oligosaccharides by infant fecal inocula. J. Agric. Food Chem..

[11] Yu ZT (2013). The principal fucosylated oligosaccharides of human milk exhibit prebiotic properties on cultured infant microbiota. Glycobiology.

[12] Yu ZT, Chen C, Newburg DS (2013). Utilization of major fucosylated and sialylated human milk oligosaccharides by isolated human gut microbes. Glycobiology.

[13] Ma L (2018). Lactational changes in the human milk oligosaccharide concentration in Chinese and Malaysian mothers’ milk. Int. Dairy J..

[14] Mäkelainen HS, Mäkivuokko HA, Salminen SJ, Rautonen NE, Ouwehand AC (2007). The effects of polydextrose and xylitol on microbial community and activity in a 4-stage colon simulator. J. Food Sci..

[15] Mäkeläinen H (2010). Synbiotic effects of GOS, PDX and Bifidobacterium lactis Bi-07 *in vitro*. International Journal of Probiotics and Prebiotics 5(4): 203-210. International Journal of Probiotics and Prebiotics.

[16] Mäkivuokko H, Nurmi J, Nurminen P, Stowell J, Rautonen N (2005). *In vitro* effects on polydextrose by colonic bacteria and caco-2 cell cyclooxygenase gene expression. Nutr. Cancer.

[17] Mäkivuokko HA, Saarinen MT, Ouwehand AC, Rautonen NE (2006). Effects of lactose on colon microbial community structure and function in a four-stage semi-continuous culture system. Biosci. Biotechnol. Biochem..

[18] Sierra C (2014). Prebiotic effect during the first year of life in healthy infants fed formula containing GOS as the only prebiotic: a multicentre, randomised, double-blind and placebo-controlled trial. Eur. J. Nutr..

[19] De Leoz ML (2015). Human milk glycomics and gut microbial genomics in infant feces show a correlation between human milk oligosaccharides and gut microbiota: a proof-of-concept study. J. Proteome Res..

[20] Verbeke KA (2015). Towards microbial fermentation metabolites as markers for health benefits of prebiotics. Nutr. Res. Rev..

[21] Davis EC, Wang M, Donovan SM (2017). The role of early life nutrition in the establishment of gastrointestinal microbial composition and function. Gut Microbes.

[22] Chichlowski M, German JB, Lebrilla CB, Mills DA (2011). The influence of milk oligosaccharides on microbiota of infants: opportunities for formulas. Annu Rev Food Sci Technol.

[23] Lewis ZT (2015). Maternal fucosyltransferase 2 status affects the gut bifidobacterial communities of breastfed infants. Microbiome.

[24] Martin R (2016). Early-Life events, including mode of delivery and type of feeding, siblings and gender, shape the developing gut microbiota. PLoS One.

[25] Korpela K, de Vos WM (2018). Early life colonization of the human gut: microbes matter everywhere. Curr. Opin. Microbiol..

[26] Wampach L (2017). Colonization and Succession within the Human Gut Microbiome by Archaea, Bacteria, and Microeukaryotes during the First Year of Life. Front. Microbiol..

[27] Bäckhed F (2015). Dynamics and stabilization of the human gut microbiome during the first year of life. Cell Host and Microbe.

[28] Guaraldi F, Salvatori G (2012). Effect of breast and formula feeding on gut microbiota shaping in newborns. Frontiers in cellular and infection microbiology.

[29] Koenig JE (2011). Succession of microbial consortia in the developing infant gut microbiome. Proc. Natl. Acad. Sci. USA.

[30] Matsuki T (2016). A key genetic factor for fucosyllactose utilization affects infant gut microbiota development. Nat Commun.

[31] Wiese M (2018). CoMiniGut-a small volume *in vitro* colon model for the screening of gut microbial fermentation processes. PeerJ.

[32] Vatanen T (2016). Variation in Microbiome LPS Immunogenicity Contributes to Autoimmunity in Humans. Cell.

[33] Asakuma S (2011). Physiology of consumption of human milk oligosaccharides by infant gut-associated bifidobacteria. J. Biol. Chem..

[34] LoCascio RG (2009). A versatile and scalable strategy for glycoprofiling bifidobacterial consumption of human milk oligosaccharides. Microb. Biotechnol..

[35] de Weerth C, Fuentes S, de Vos WM (2013). Crying in infants: On the possible role of intestinal microbiota in the development of colic. Gut Microbes.

[36] Abrahamsson TR (2012). Low diversity of the gut microbiota in infants with atopic eczema. J. Allergy Clin. Immunol..

[37] de Weerth C (2017). Do bacteria shape our development? Crosstalk between intestinal microbiota and HPA axis. Neurosci. Biobehav. Rev..

[38] De Weerth C, Fuentes S, Puylaert P, De Vos WM (2013). Intestinal microbiota of infants with colic: Development and specific signatures. Pediatrics.

[39] Nylund L (2015). Severity of atopic disease inversely correlates with intestinal microbiota diversity and butyrate-producing bacteria. Allergy.

[40] Turroni F (2018). Glycan Utilization and Cross-Feeding Activities by Bifidobacteria. Trends Microbiol..

[41] Turroni F (2016). Deciphering bifidobacterial-mediated metabolic interactions and their impact on gut microbiota by a multi-omics approach. ISME Journal.

[42] Elison E (2016). Oral supplementation of healthy adults with 2′-O-fucosyllactose and lacto-N-neotetraose is well tolerated and shifts the intestinal microbiota. Br. J. Nutr..

[43] Thongaram T, Hoeflinger JL, Chow J, Miller MJ (2017). Human milk oligosaccharide consumption by probiotic and human-associated bifidobacteria and lactobacilli. J. Dairy Sci..

[44] Bonk F, Popp D, Harms H, Centler F (2018). PCR-based quantification of taxa-specific abundances in microbial communities: Quantifying and avoiding common pitfalls. J. Microbiol. Methods.

[45] Ciofu O, Hansen CR, Høiby N (2013). Respiratory bacterial infections in cystic fibrosis. Curr. Opin. Pulm. Med..

[46] Sadikot RT, Blackwell TS, Christman JW, Prince AS (2005). Pathogen-host interactions in pseudomonas aeruginosa pneumonia. Am. J. Respir. Crit. Care Med..

[47] Zheng H (2016). Altered Gut Microbiota Composition Associated with Eczema in Infants. PLoS One.

[48] Duncan SH, Barcenilla A, Stewart CS, Pryde SE, Flint HJ (2002). Acetate utilization and butyryl coenzyme A (CoA):acetate-CoA transferase in butyrate-producing bacteria from the human large intestine. Appl. Environ. Microbiol..

[49] Marcobal A (2010). Consumption of human milk oligosaccharides by gut-related microbes. J. Agric. Food Chem..

[50] Timmerman HM (2017). Intestinal colonisation patterns in breastfed and formula-fed infants during the first 12 weeks of life reveal sequential microbiota signatures. Sci. Rep..

[51] Wang M (2015). Fecal microbiota composition of breast-fed infants is correlated with human milk oligosaccharides consumed. J. Pediatr. Gastroenterol. Nutr..

[52] Dubin, K. & Pamer, E. G. Enterococci and Their Interactions with the Intestinal Microbiome. *Microbiology**Spectrum* **5**, 10.1128/microbiolspec.BAD-0014-2016 (2014). 10.1128/microbiolspec.bad-0014-2016 PMC5691600 29125098

[53] Wang S, Hibberd ML, Pettersson S, Lee YK (2014). Enterococcus faecalis from healthy infants modulates inflammation through MAPK signaling pathways. PLoS One.

[54] Wang S, Ng LH, Chow WL, Lee YK (2008). Infant intestinal Enterococcus faecalis down-regulates inflammatory responses in human intestinal cell lines. World J. Gastroenterol..

[55] Fallani M (2010). Intestinal microbiota of 6-week-old infants across Europe: Geographic influence beyond delivery mode, breast-feeding, and antibiotics. J. Pediatr. Gastroenterol. Nutr..

[56] Gomez-Llorente C (2013). Three main factors define changes in fecal microbiota associated with feeding modality in infants. J. Pediatr. Gastroenterol. Nutr..

[57] Thompson AL, Monteagudo-Mera A, Cadenas MB, Lampl ML, Azcarate-Peril MA (2015). Milk- and solid-feeding practices and daycare attendance are associated with differences in bacterial diversity, predominant communities, and metabolic and immune function of the infant gut microbiome. *Frontiers in Cellular and Infection*. Microbiology.

[58] Vallès Y (2014). Microbial Succession in the Gut: Directional Trends of Taxonomic and Functional Change in a Birth Cohort of Spanish Infants. PLoS Genet..

[59] Le Huërou-Luron I, Blat S, Boudry G (2010). Breast- v. formula-feeding: impacts on the digestive tract and immediate and long-term health effects. Nutr. Res. Rev..

[60] Ogawa K, Ben RA, Pons S, de Paolo MIL, Fernández LB (1992). Volatile fatty acids, lactic acid, and pH in the stools of breast-fed and bottle-fed infants. J. Pediatr. Gastroenterol. Nutr..

[61] Medina Daniel, Pinto Francisco, Ovalle Aline, Thomson Pamela, Garrido Daniel (2017). Prebiotics Mediate Microbial Interactions in a Consortium of the Infant Gut Microbiome. International Journal of Molecular Sciences.

[62] Parrett AM, Edwards CA (1997). *In vitro* fermentation of carbohydrate by breast fed and formula fed infants. Arch. Dis. Child..

[63] Pham VT, Lacroix C, Braegger CP, Chassard C (2017). Lactate-utilizing community is associated with gut microbiota dysbiosis in colicky infants. Sci. Rep..

[64] Binder, H. J. In *Annu*. *Rev*. *Physiol*. Vol. 72 297–313 (2009). 10.1146/annurev-physiol-021909-135817 20148677

[65] Wang J (2017). Relative fermentation of oligosaccharides from human milk and plants by gut microbes. Eur. Food Res. Technol..

[66] Fan P (2017). Roles of biogenic amines in intestinal signaling. Current Protein and Peptide Science.

[67] Mäkivuokko H (2007). The effect of cocoa and polydextrose on bacterial fermentation in gastrointestinal tract simulations. Biosci. Biotechnol. Biochem..

[68] Mäkeläinen H (2010). Xylo-oligosaccharides enhance the growth of bifidobacteria and Bifidobacterium lactis in a simulated colon model. Benef Microbes.

[69] Raza GS (2017). Polydextrose changes the gut microbiome and attenuates fasting triglyceride and cholesterol levels in Western diet fed mice. Sci. Rep..

[70] Caporaso JG (2010). QIIME allows analysis of high-throughput community sequencing data. Nat. Methods.

[71] DeSantis TZ (2006). Greengenes, a chimera-checked 16S rRNA gene database and workbench compatible with ARB. Appl. Environ. Microbiol..

[72] Ouwehand AC, Tiihonen K, Saarinen M, Putaala H, Rautonen N (2009). Influence of a combination of Lactobacillus acidophilus NCFM and lactitol on healthy elderly: intestinal and immune parameters. Br. J. Nutr..

[73] Saarinen MT (2002). Determination of biogenic amines as dansyl derivatives in intestinal digesta and feces by reversed phase HPLC. Chromatographia.

[74] Faith DP (1992). Conservation evaluation and phylogenetic diversity. Biol. Conserv..

[75] Benjamini Y, Hochberg Y (1995). Controlling the False Discovery Rate: A Practical and Powerful Approach to Multiple Testing. Journal of the Royal Statistical Society. Series B (Methodological).

[76] Lozupone C, Knight R (2005). UniFrac: a new phylogenetic method for comparing microbial communities. Appl. Environ. Microbiol..

[77] Team, R. D. C. R: A language and environment for statistical computing Vienna Austria, http://www.R-project.org (2008.).

[78] Wickham, H. *ggplot2: Elegant Graphics for Data Analysis*. (Springer Publishing Company, Incorporated, 2009).

[79] Brunner, E., Domhof, S. & Langer, F. *Nonparametric analysis of longitudinal data in factorial experiments*. (New York, 2002).

[80] Noguchi K, Gel YR, Brunner E, Konietschke F (2012). nparLD: An R Software Package for the Nonparametric Analysis of Longitudinal Data in Factorial Experiments. Journal of Statistical Software.

