# Supplementary material for: The effect of 2′-fucosyllactose on simulated infant gut microbiome and metabolites; a pilot study in comparison to GOS and lactose
Source: Sci Rep. 2019 Sep 13;9:13232. doi: 10.1038/s41598-019-49497-z (PMC6744565; doi:10.1038/s41598-019-49497-z)
Supplement: Supplementary file 1 — Supplementary tables and figures [file 41598_2019_49497_MOESM1_ESM.docx]

# The effect of 2’-fucosyllactose on simulated infant gut microbiome and metabolites; a pilot study in comparison to GOS and lactose

**Krista Salli, Heli Anglenius, Johanna Hirvonen, Ashley A. Hibberd, Ilmari Ahonen, Markku T. Saarinen, Kirsti Tiihonen, Johanna Maukonen & Arthur C. Ouwehand**

**Supplementary Figures and Tables:**

**Supplementary Table S1** Faecal inocula metabolite composition, total bacteria by flow cytometry and total bifidobacteria by qPCR.

|  |  | Faecal inocula sample donor | | | | | | | | |
| --- | --- | --- | --- | --- | --- | --- | --- | --- | --- | --- |
| Unit | Metabolite | 001 | 002 | 003 | 012 | 011 | 006 | 008 | 013 | 004 |
| µmol/ml | Acetic acid | 56.5 | 21.8 | 43.6 | 20.5 | 53.7 | 66.0 | 51.1 | 36.8 | 51.1 |
|  | Propionic acid | 1.4 | 4.7 | 0.8 | 2.0 | 1.0 | 3.4 | 7.3 | 0.7 | 6.8 |
|  | Butyric acid | ND | 1.1 | 1.0 | 4.6 | <0.2 | 0.4 | 3.4 | 1.4 | 0.3 |
|  | Valeric acid | ND | ND | ND | ND | ND | ND | ND | ND | ND |
|  | Lactic acid | 27.1 | 18.3 | 31.4 | 19.8 | 30.9 | 49.2 | 12.6 | 15.7 | 22.9 |
|  | Isobutyric acid | ND | ND | <0.1 | 0.9 | ND | 0.2 | 0.3 | <0.2 | 0.2 |
|  | 2-methylbutyric acid | ND | ND | ND | 0.6 | ND | <0.1 | <0.2 | ND | 0.1 |
|  | Isovaleric acid | ND | ND | ND | <0.2 | <0.2 | 0.1 | <0.2 | ND | 0.2 |
|  | Methylamine | 849 | 255 | 599 | 313 | 1506 | 972 | 1010 | 246 | 1184 |
|  | Ethylamine | 1159 | 57 | 516 | <40 | <40 | 1513 | <40 | <40 | 1194 |
|  | Tryptamine | <10 | 11.7 | <10 | <10 | <10 | 246.2 | <10 | <10 | 12.9 |
|  | β-Phenyl-ethylamine | 30.6 | 61.7 | <15 | 29.3 | 34.7 | 20.3 | <15 | <15 | 22.6 |
|  | 2-methyl-butylamine | <20 | <20 | 61.7 | <20 | <20 | <20 | <20 | <20 | <20 |
|  | Putrescine | 473 | 535 | 824 | 725 | 476 | 676 | 170 | 24 | 561 |
|  | Cadaverine | 946 | 1660 | 840 | 2820 | 54 | 85 | 139 | 25 | 140 |
|  | Histamine | <3 | <3 | 3.2 | <3 | 19.7 | 6.8 | <3 | 4.5 | 12.4 |
|  | Tyramine | 907 | 644 | 499 | 845 | 681 | 1097 | 17 | 15 | 731 |
|  | Spermidine | 23.2 | 5.4 | 48.2 | 29.7 | 10.7 | 53.0 | 33.7 | 12.4 | 13.0 |
|  | Spermine | 5.0 | 4.9 | 6.4 | 4.5 | 4.0 | 4.7 | 7.1 | 3.4 | 6.6 |
| log10/ml | Total bacteria by flow cytometry | 9.2 | 9.1 | 9.5 | 9.4 | 10.0 | 10.0 | 9.9 | 9.5 | 10.0 |
| log10/ml | Total bifidobacteria by qPCR | 9.9 | BDL | 9.3 | BDL | 10.0 | 9.1 | 10.0 | 9.2 | 9.7 |

ND=not detected, BDL=below detection limit

**Supplementary Table S2.** P-values for the treatment effect on microbial metabolite level and production profile. The treatments are compared against the control in the combined data from the simulations from the nine donors, and separately within groups. The breast-fed group refers to simulations with breast-fed donors 001, 002, 003, 004 and 006 and formula-fed group refers to simulations with formula-fed donors 008, 011, 012, and 013. The 2’-fucosyllactose (2’-FL) fast-fermenting group refers to simulations with donors 004, 008 and 011, and slow-fermenting group refers to simulations with donors 001, 002, 003, 006, 012, and 013. The obtained p-values were FDR-corrected. SCFA = short-chain fatty acids, BCFA = branched-chain fatty acids, BA= biogenic amines, GOS = galacto-oligosaccharides.

| Data used | Measurement | | 2’-FL | | Lactose | | GOS | |
| --- | --- | --- | --- | --- | --- | --- | --- | --- |
|  |  |  | Level | Profile | Level | Profile | Level | Profile |
| All data | | SCFA and lactic acid sum | **0.014** | 0.675 | **<0.001** | **0.001** | **<0.001** | **0.009** |
|  |  | BCFA sum | 0.453 | 0.225 | **0.007** | **<0.001** | **0.019** | **0.001** |
|  |  | BA sum | 0.652 | 0.225 | 0.453 | 0.129 | 0.665 | 0.225 |
| Breast fed | | SCFA and lactic acid sum | 0.294 | 0.302 | **<0.001** | **0.009** | **<0.001** | **0.013** |
|  |  | BCFA sum | 0.296 | 0.447 | 0.096 | **0.025** | **0.074** | **0.012** |
|  |  | BA sum | 0.184 | **0.010** | **0.047** | 0.225 | 0.413 | 0.725 |
| Formula fed | | SCFA and lactic acid sum | **0.002** | 0.225 | **<0.001** | 0.166 | **<0.001** | 0.242 |
|  |  | BCFA sum | 0.853 | 0.630 | **0.009** | **0.012** | 0.074 | **0.037** |
|  |  | BA sum | 0.211 | 0.374 | 0.649 | 0.341 | 0.968 | **0.020** |
| 2'-FL Fast | | SCFA and lactic acid sum | **<0.001** | 0.267 | **<0.001** | **<0.001** | **<0.001** | 0.251 |
|  |  | BCFA sum | **0.004** | 0.225 | **0.007** | 0.102 | **0.001** | 0.322 |
|  |  | BA sum | 0.817 | 0.492 | 0.968 | 0.630 | 0.858 | 0.322 |
| 2'-FL Slow | | SCFA and lactic acid sum | 0.126 | 0.659 | **<0.001** | **0.013** | **<0.001** | **0.007** |
|  |  | BCFA sum | 0.852 | 0.675 | 0.086 | **0.009** | 0.146 | **0.002** |
|  |  | BA sum | 0.464 | 0.225 | 0.430 | 0.217 | 0.453 | 0.265 |

**Supplementary Table S3.** Phylum- and genus-level microbiota changes among treatment groups between breast-fed and formula-fed infants with all vessels combined from the individual treatment. The breast-fed group refers to simulations with breast-fed donors 001, 002, 003, 004 and 006 and formula-fed group refers to simulations with formula-fed donors 008, 011, 012, and 013. 2’-FL = 2’-fucosyllactose, GOS = galacto-oligosaccharides.

|  | Breast-fed | | | | | Formula-fed | | | | |
| --- | --- | --- | --- | --- | --- | --- | --- | --- | --- | --- |
| Taxon | Abundance, % (mean±SD) | | | | Overall p-value  (FDR adj)^1^ | Abundance, % (mean±SD) | | | | Overall p-value  (FDR adj)^1^ |
|  | Control | 2’-FL | Lactose | GOS |  | Control | 2’-FL | Lactose | GOS |  |
| Actinobacteria | 19.12 ± 13.7 | 31.84 ± 23.0 | 29.45 ± 27.3 | 31.40 ± 25.3 | 0.4 | 22.51 ± 12.0 | 20.48 ± 13.3 | 25.82 ± 17.6 | 28.63 ± 18.1 | 0.8 |
| *Aeromicrobium* | 0.20 ± 0.34 | 0.14 ± 0.25 | **0.02 ± 0.05** | **0.02 ± 0.06** | 0.02 |  |  |  |  |  |
| *Cellulosimicrobium* | 0.24 ± 0.14 | 0.27 ± 0.26 | **0.07 ± 0.07** | **0.03 ± 0.04** | <0.001 |  |  |  |  |  |
| Bacteroidetes | 11.95 ± 16.2 | **0.67 ± 1.6** | **0.46 ± 0.96** | 2.35 ± 4.1 | 0.03 | 13.39 ± 18.3 | 5.47 ± 8.6 | 1.58 ± 1.6 | 1.34 ± 2.9 | 0.8 |
| *Bacteroides* | 8.94 ± 12.7 | **0.62 ± 1.6** | 0.45 ± 0.95 | 2.3 ± 4.0 | 0.03 |  |  |  |  |  |
| Firmicutes | 33.57 ± 12.3 | 39.84 ± 15.5 | **60.09 ± 25.3** | **55.03 ± 19.1** | 0.001 | 36.52 ± 11.1 | **59.94 ± 15.8** | **60.09 ± 19.8** | **59.84 ± 21.0** | 0.003 |
| *Clostridiaceae_uncl* |  |  |  |  |  | 3.15 ± 5.1 | 4.52 ± 6.1 | 0.63 ± 0.10 | 0.83 ± 1.7 | 0.08 |
| *Enterococcus* | 3.52 ± 3.9 | **7.32 ± 6.4** | 7.70 ± 15.3 | 3.64 ± 7.5 | 0.03 |  |  |  |  |  |
| *Lachnospiraceae_uncl* | 0.34 ± 0.45 | 0.35 ± 0.64 | **0.05 ± 0.09** | **0.05 ± 0.02** | 0.007 | 0.75 ± 1.1 | 0.43 ± 0.49 | 0.30 ± 0.56 | **0.17 ± 0.30** | 0.08 |
| *Lactobacillus* | 6.44 ± 7.4 | 9.83 ± 6.7 | 24.02 ± 30.2 | **25.47 ± 26.4** | 0.05 | 11.82 ± 10.6 | 21.97 ± 23.9 | **40.31 ± 24.8** | **35.90 ± 26.2** | 0.01 |
| *Rummeliibacillus* |  |  |  |  |  | 0.23 ± 0.38 | 0.27 ± 0.61 | 0.03 ± 0.04 | **0.04 ± 0.03** | 0.09 |
| *Tissierella_Soehngenia* | 1.16 ± 2.8 | 1.06 ± 2.4 | 0.26 ± 1.1 | **0.07 ± 0.29** | 0.06 |  |  |  |  |  |
| *Coprococcus* |  |  |  |  |  | 0.10 ± 0.21 | **1.29 ± 2.0** | 0.74 ± 1.6 | 0.36 ± 0.80 | 0.09 |
| Proteobacteria | 35.35 ± 18.9 | 27.65 ± 16.7 | **10.00 ± 11.4** | **11.21 ± 11.8** | <0.001 | 25.66 ± 14.6 | **13.63 ± 11.7** | **12.38 ± 8.1** | **10.10 ± 7.3** | 0.005 |
| *Achromobacter* | 0.70 ± 0.44 | **0.32 ± 0.40** | **0.08 ± 0.16** | **0.05 ± 0.07** | <0.001 | 0.89 ± 0.84 | **0.23 ± 0.21** | **0.10 ± 0.12** | **0.08 ± 0.08** | <0.001 |
| *Agrobacterium* | 0.34 ± 0.53 | 0.20 ± 0.24 | **0.05 ± 0.07** | **0.02 ± 0.03** | <0.001 | 0.31 ± 0.36 | **0.13 ± 0.16** | **0.12 ± 0.16** | **0.02 ± 0.02** | <0.001 |
| *Citrobacter* | 0.16 ± 0.12 | **0.06 ± 0.05** | **0.05 ± 0.14** | **0.03 ± 0.05** | <0.001 |  |  |  |  |  |
| *Enterobacteriaceae* spp. | 0.20 ± 0.17 | 0.20 ± 0.12 | **0.07 ± 0.08** | **0.09 ± 0.10** | 0.002 |  |  |  |  |  |
| *Enterobacteriaceae_uncl* | 32.80 ± 18.6 | 26.26 ± 16.3 | **9.47 ± 10.4** | **10.66 ± 11.3** | <0.001 | 22.02 ± 14.8 | 11.95 ± 10.8 | 11.21 ± 8.3 | **9.51 ± 7.3** | 0.09 |
| *Pseudomonadaceae_uncl* | 0.18 ± 0.42 | 0.16 ± 0.26 | 0.02 ± 0.03 | 0.08 ± 0.29 | 0.03 |  |  |  |  |  |
| *Pseudomonas* | 0.56 ± 0.67 | **0.25 ± 0.47** | **0.18 ± 0.68** | **0.20 ± 0.40** | <0.001 | 1.29 ± 1.8 | 0.50 ± 0.82 | **0.52 ± 1.1** | **0.10 ± 0.24** | <0.001 |
| Verrucomicrobia | 0.0 ± 0.0 | 0.01 ± 0.01 | 0.0 ± 0.0 | 0.1 ± 0.1 | 0.4 | 1.92 ± 4.4 | 0.47 ± 1.5 | 0.12 ± 0.30 | 0.09 ± 0.30 | 0.9 |

^1^Kruskal-Wallis test with Benjamini-Hochberg false discovery rate (FDR) adjustment were conducted for main effect of treatment, and only genera with FDR<0.1 are shown. Bolded treatment means differ from Control p<0.05 (Steel With Control post hoc test).

**Supplementary Table S4**. Phylum- and genus-level microbiota changes among treatment groups between fast and slow 2’-fucosyllactose (2’-FL) fermenters within the 2’-FL group with all vessels combined from the individual treatment. The fast-fermenting group refers to simulations with donors 004, 008 and 011, and slow-fermenting group refers to simulations with donors 001, 002, 003, 006, 012, and 013. GOS = galacto-oligosaccharides.

| Taxon | 2'-FL Fast | | | | | 2'-FL Slow | | | | |
| --- | --- | --- | --- | --- | --- | --- | --- | --- | --- | --- |
|  | Abundance, % (mean±SD) Overall p-value | | | | Overall p-value  (FDR adj)^1^ | Abundance, % (mean±SD) | | | | Overall p-value  (FDR adj)^1^ |
|  | Control | 2’-FL | Lactose | GOS |  | Control | 2’-FL | Lactose | GOS |  |
| Actinobacteria | 26.45 ± 12.7 | 36.94 ± 20.2 | 25.91 ± 16.7 | 37.61 ± 17.4 | 0.3 | 18.33 ± 12.4 | 21.71 ± 18.0 | 28.65 ± 25.8 | 26.45 ± 23.6 | 0.8 |
| *Aeromicrobium* |  |  |  |  |  | 0.29 ± 0.49 | 0.19 ± 0.34 | **0.03 ± 0.06** | **0.02 ± 0.08** | 0.004 |
| *Cellulosimicrobium* |  |  |  |  |  | 0.29 ± 0.24 | 0.26 ± 0.24 | **0.08 ± 0.07** | **0.04 ± 0.04** | <0.001 |
| Bacteroidetes | 13.59 ± 18.2 | 4.10 ± 5.4 | 1.89 ± 3.3 | 2.69 ± 3.6 | 0.9 | 12.22 ± 16.9 | 2.15 ± 6.6 | 0.55 ± 1.4 | 1.51 ± 3.6 | 0.13 |
| Firmicutes | 36.55 ± 13.0 | **54.52 ± 16.9** | **64.49 ± 19.4** | 51.14 ± 17.9 | 0.02 | 34.30 ± 11.3 | 45.90 ± 18.8 | **58.07 ± 24.0** | **60.18 ± 20.4** | <0.001 |
| *Clostridiaceae_uncl* |  |  |  |  |  | 1.37 ± 1.7 | 4.44 ± 5.3 | 0.57 ± 1.0 | 0.57 ± 1.5 | 0.004 |
| *Enterococcus* |  |  |  |  |  | 4.62 ± 5.2 | 6.71 ± 6.0 | 1.78 ± 0.34 | 3.82 ± 7.0 | 0.002 |
| *Lachnospiraceae_uncl* |  |  |  |  |  | 0.42 ± 0.57 | 0.33 ± 0.61 | **0.06 ± 0.12** | **0.03 ± 0.08** | <0.001 |
| *Lactobacillus* |  |  |  |  |  | 8.06 ± 9.3 | 10.91 ± 11.5 | **31.69 ± 30.6** | **32.64 ± 29.3** | 0.001 |
| *Rummeliibacillus* |  |  |  |  |  | 0.26 ± 0.43 | 0.21 ± 0.51 | **0.03 ± 0.09** | 0.06 ± 0.14 | 0.002 |
| *Tissierella_Soehngenia* |  |  |  |  |  | 1.91 ± 3.2 | 1.19 ± 2.4 | **0.21 ± 0.96** | **0.09 ± 0.32** | 0.02 |
| Proteobacteria | 20.32 ± 13.7 | **4.34 ± 4.0** | **7.62 ± 4.7** | **8.43 ± 9.1** | 0.004 | 35.15 ± 17.3 | 29.96 ± 12.7 | **12.68 ± 11.4** | **11.85 ± 10.3** | <0.001 |
| *Achromobacter* | 0.67 ± 0.49 | **0.13 ± 0.13** | **0.12 ± 0.13** | **0.08 ± 0.09** | 0.009 | 0.85 ± 0.71 | **0.36 ± 0.37** | **0.08 ± 0.15** | **0.06 ± 0.07** | <0.001 |
| *Agrobacterium* | 0.43 ± 0.51 | 0.12 ± 0.16 | 0.14 ± 0.26 | **0.02 ± 0.02** | 0.02 | 0.28 ± 0.43 | 0.19 ± 0.22 | **0.06 ± 0.06** | **0.02 ± 0.03** | <0.001 |
| *Citrobacter* |  |  |  |  |  | 0.28 ± 0.32 | 0.36 ± 0.54 | **0.10 ± 0.18** | **0.08 ± 0.12** | <0.001 |
| *Enterobacteriaceae* spp. | 0.12 ± 0.12 | **0.03 ± 0.04** | 0.06 ± 0.05 | 0.06 ± 0.07 | 0.07 | 0.21 ± 0.17 | 0.21 ± 0.09 | **0.08 ± 0.07** | **0.09 ± 0.09** | <0.001 |
| *Enterobacteriaceae_uncl* | 16.63 ± 13.2 | **3.67 ± 3.3** | **6.60 ± 4.6** | 7.92 ± 9.0 | 0.02 | 32.36 ± 17.3 | 28.02 ± 12.7 | **11.94 ± 10.6** | **11.27 ± 9.9** | <0.001 |
| *Pseudomonadaceae_uncl* |  |  |  |  |  | 0.17 ± 0.37 | 0.16 ± 0.25 | **0.03 ± 0.04** | **0.09 ± 0.27** | 0.002 |
| *Pseudomonas* | 1.55 ± 2.2 | **0.14 ± 0.22** | **0.43 ± 0.90** | **0.12 ± 0.27** | 0.02 | 0.64 ± 0.75 | 0.47 ± 0.76 | **0.29 ± 0.91** | **0.17 ± 0.37** | <0.001 |
| Verrucomicrobia | 3.07 ± 5.4 | 0.09 ± 0.20 | 0.09 ± 0.20 | 0.12 ± 0.30 | 0.9 | 0.1 ± 0.1 | 0.28 ± 1.3 | 0.04 ± 0.20 | 0.1 ± 0.1 | 0.8 |

^1^Kruskal-Wallis test with Benjamini-Hochberg false discovery rate (FDR) adjustment were conducted for main effect of treatment, and only genera with FDR<0.1 are shown. Bolded treatment means differ from Control p<0.05 (Steel With Control post hoc test).

**
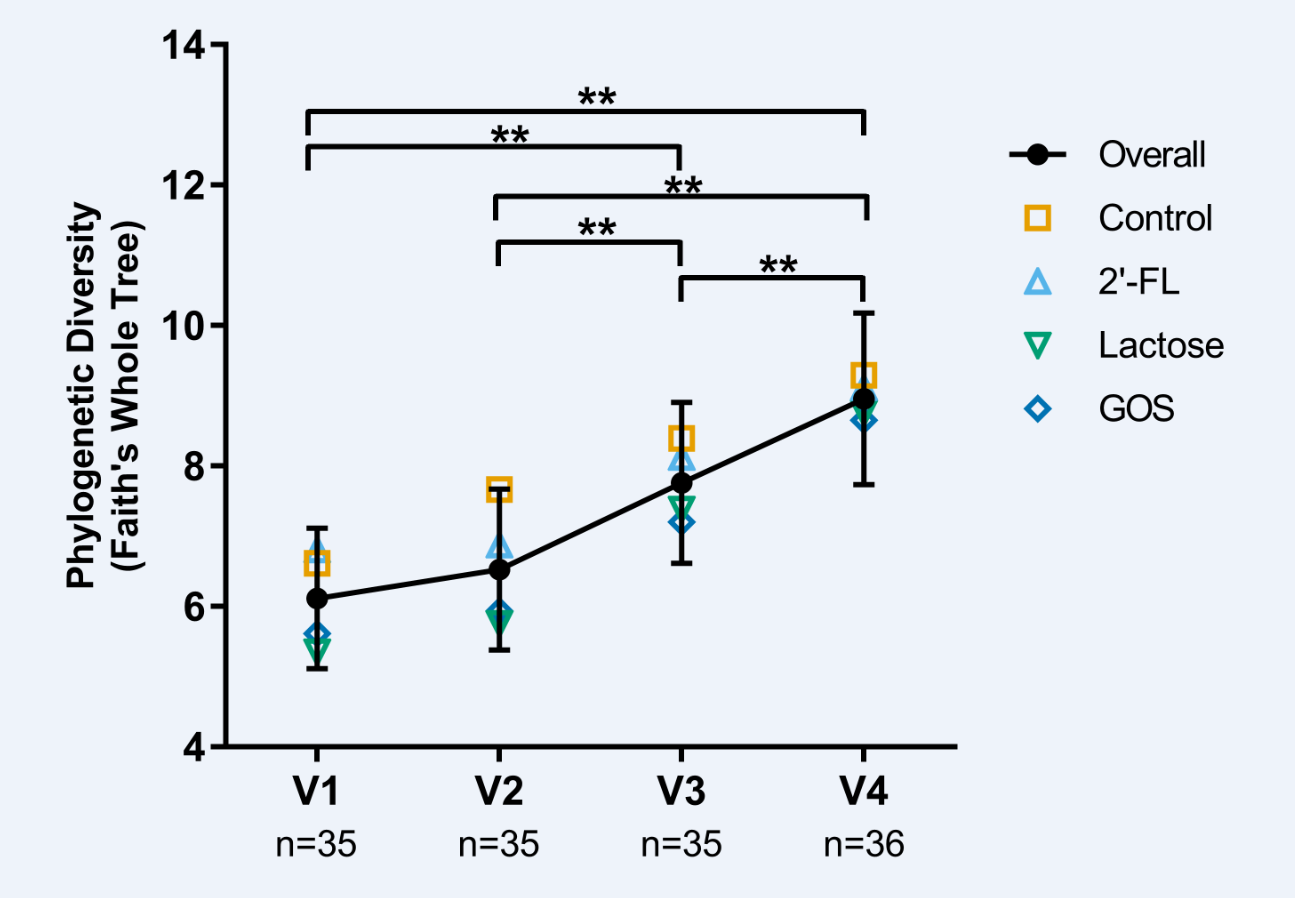
**

**Supplementary Figure S1** Alpha diversity (Faith’s Whole Tree metric) from Vessel 1 to Vessel 4. 2’-FL = 2’-fucosyllactose, GOS = galacto-oligosaccharides.


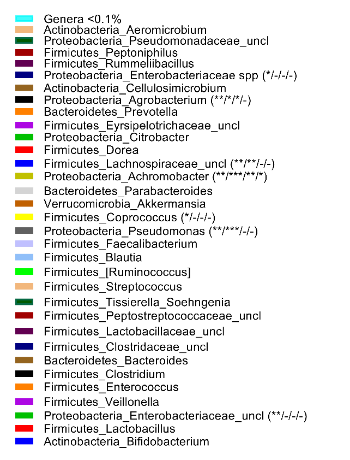
 **
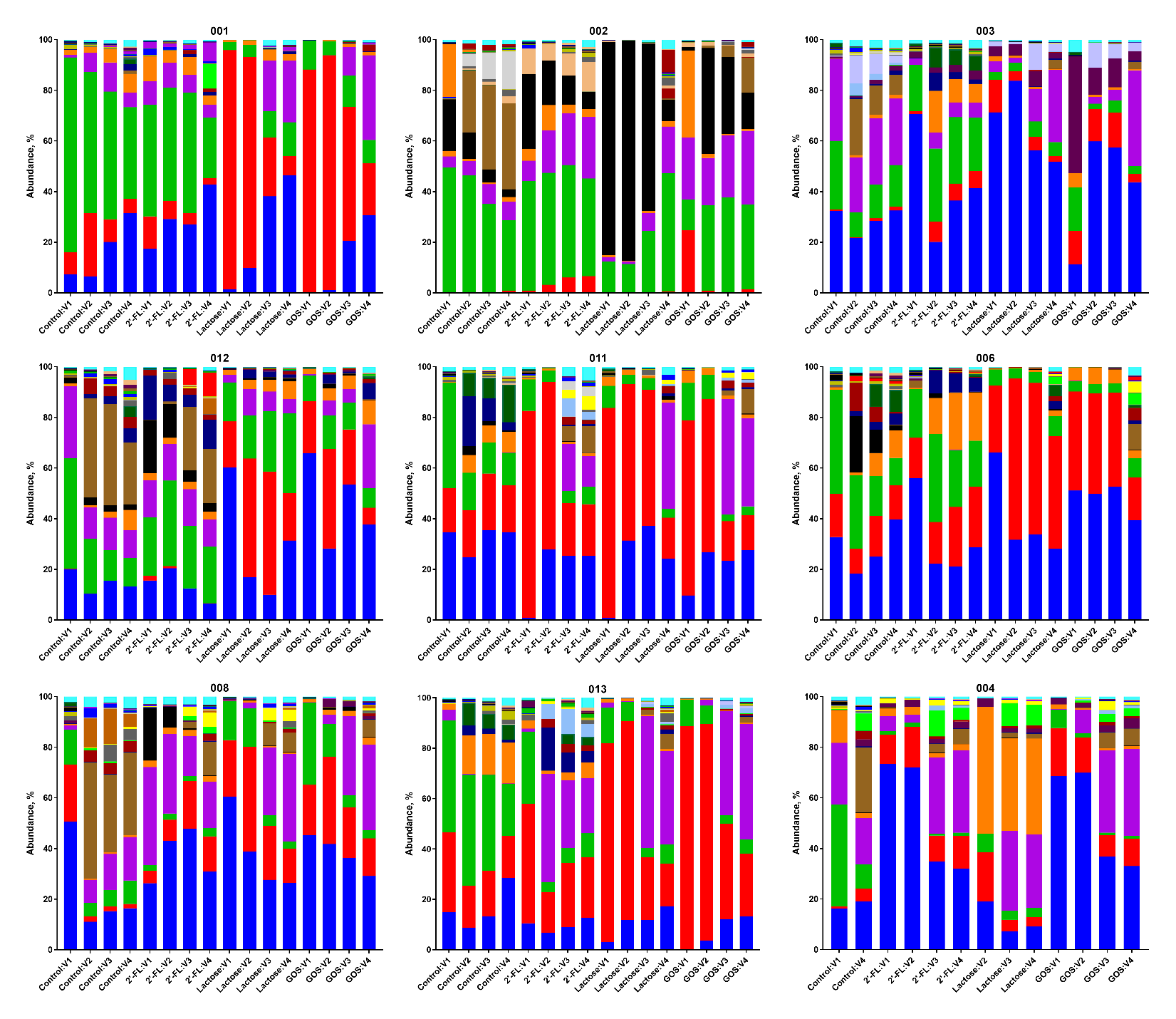
**

**Supplementary Figure S2.**

Relative abundance of bacteria at genus level of taxonomy in different simulation vessels and different treatments. 2’-FL = 2’-fucosyllactose, GOS = galacto-oligosaccharides. Significant differences among treatment groups for individual vessels are denoted (***p<0.001, **p<0.01, *p<0.05) in order of vessel (V1/V2/V3/V4) following taxa names. Kruskal-Wallis test with Benjamini-Hochberg false discovery rate (FDR) adjustment.


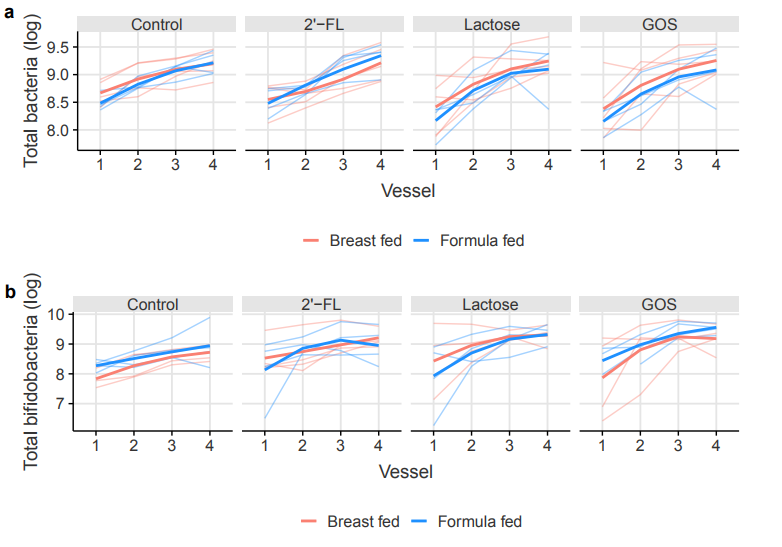

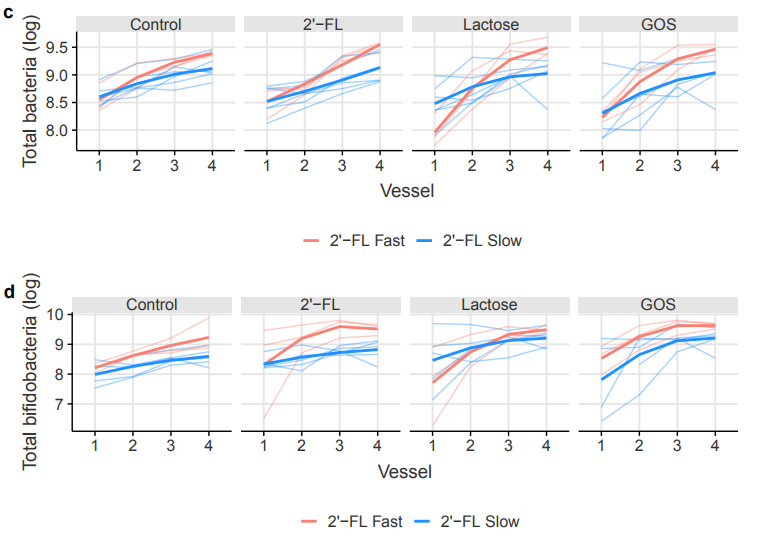


**Supplementary Figure S3.** Bold lines show smoothened averages of (a) total bacteria measured by flow cytometry and (b) total bifidobacteria measured by qPCR, dividing simulations according breast-fed group referring to simulations with breast-fed donors 001, 002, 003, 004 and 006 and formula-fed group referring to simulations with formula-fed donors 008, 011, 012, and 013; (c) total bacteria measured by flow cytometry and (d) total bifidobacteria measured by qPCR dividing simulations according 2’-fucosyllactose (2’-FL) fast-fermenting group referring to simulations with donors 004, 008 and 011, or slow-fermenting group referring to simulations with donors 001, 002, 003, 006, 012, and 013. Narrow lines show individual simulations, colours indicate the grouping. GOS= galacto-oligosaccharides.


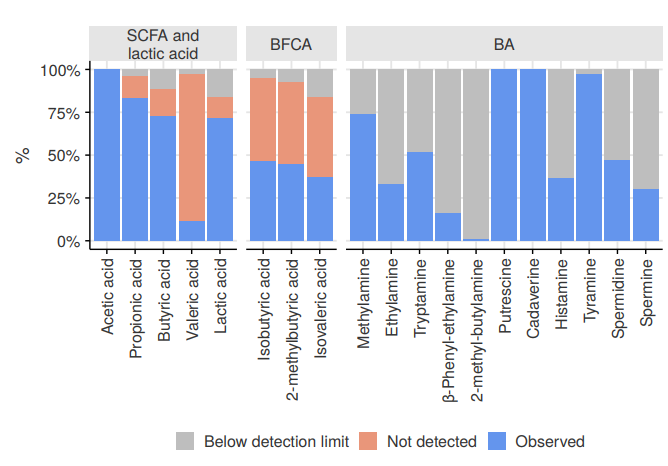


**Supplementary Figure S4.** Metabolite data distribution from all the simulations and all the vessels. SCFA = short-chain fatty acids, BCFA = branched-chain fatty acids, BA = biogenic amines.


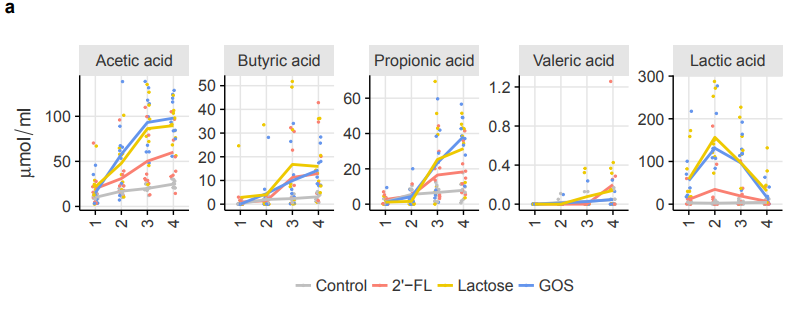


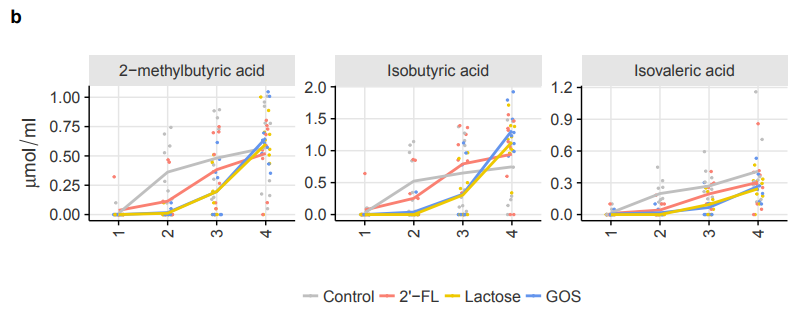


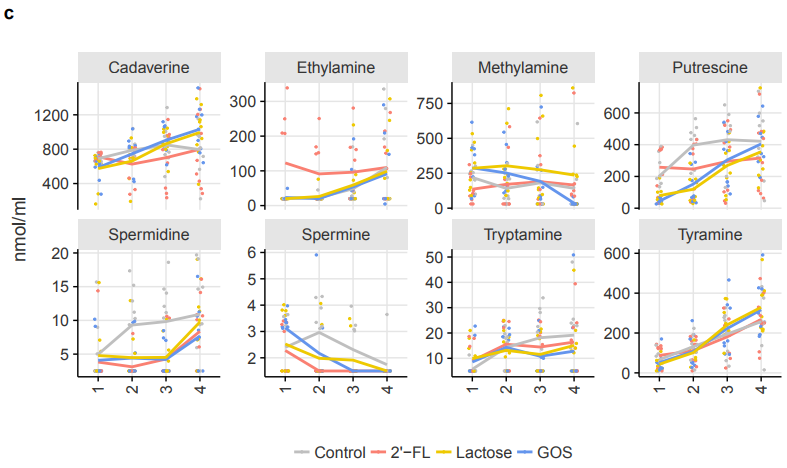


**Supplementary Figure S5.** Smoothened averages of treatment effect on separate metabolites for all simulations from the nine donors combined. (a) short chain fatty acids and lactic acid, (b) branched chain fatty acids, and (c) biogenic amines. The dots are measurements from individual simulations. 2’-FL = 2’-fucosyllactose, GOS= galacto-oligosaccharides.

(a)


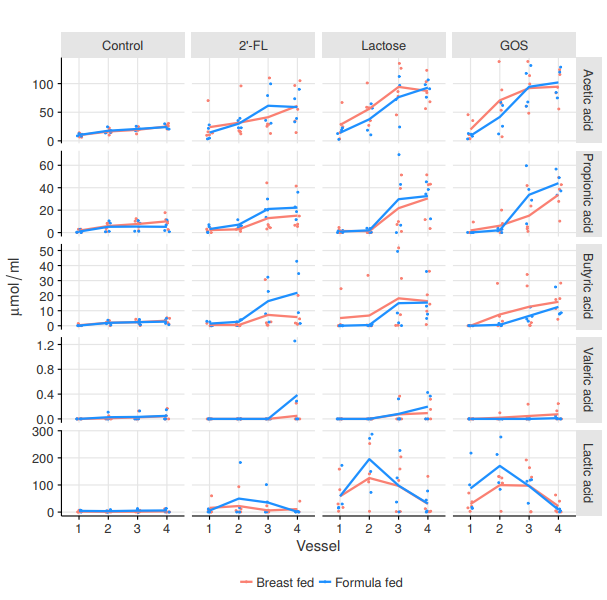


(b)


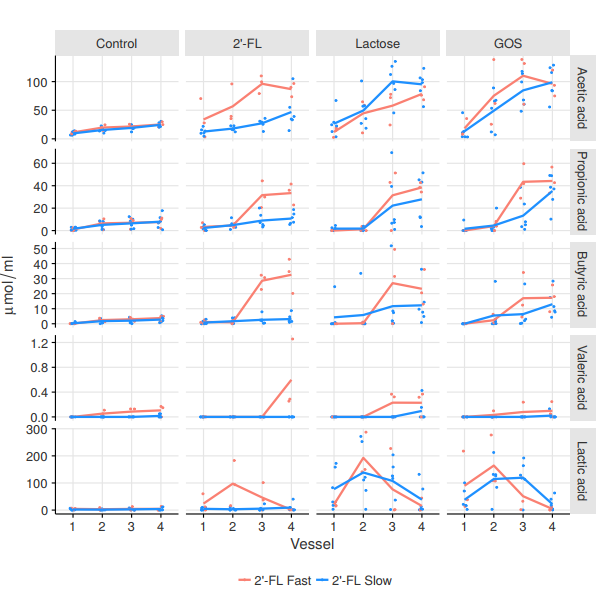


**Supplementary Figure S6.** Smoothened averages of short-chain fatty acids and lactic acid measured (a) from breast-fed group referring to simulations with breast-fed donors 001, 002, 003, 004 and 006 and formula-fed group referring to simulations with formula-fed donors 008, 011, 012, and 013 and (b) 2’-fucosyllactose (2’-FL) fast-fermenting group referring to simulations with donors 004, 008 and 011, or slow-fermenting group referring to simulations with donors 001, 002, 003, 006, 012, and 013. showing the effect of the treatment. The dots are measurements from individual simulations. GOS= galacto-oligosaccharides.
